# Endovascular therapy for superior vena cava syndrome: A systematic review and meta-analysis

**DOI:** 10.1016/j.eclinm.2021.100970

**Published:** 2021-06-28

**Authors:** Abdul Hussain Azizi, Irfan Shafi, Matthew Zhao, Saurav Chatterjee, Stephanie Clare Roth, Maninder Singh, Vladimir Lakhter, Riyaz Bashir

**Affiliations:** a Department of Medicine, Lewis Katz School of Medicine at Temple University, Philadelphia, PA, United States; b Department of Internal Medicine, Wayne State University/Detroit Medical Center, Detroit, MI, United States; c Department of Medicine, David Geffen School of Medicine at UCLA, Los Angeles, CA, United States; d Division of Cardiology, Department of Medicine, Northshore-LIJ Hospitals of Northwell Health, and Assistant Professor of Medicine, Zucker School of Medicine, New York, NY, United States; e Ginsburg Health Sciences Library, Temple University, Philadelphia, PA, United States; f Division of Cardiovascular Disease, Lewis Katz School of Medicine at Temple University, Philadelphia, PA, United States

**Keywords:** Superior vena cava syndrome, Endovascular therapy, Catheter-directed thrombolysis, Angioplasty, Stents

## Abstract

**Background:**

Superior vena cava (SVC) syndrome is caused by the obstruction of the SVC and can result in significant morbidity and mortality. In contemporary practice, endovascular therapy (ET) has become the standard of care for a majority of these patients. This study is a systematic review and meta-analysis of the available literature to assess technical success, restenosis, and recurrence of SVC syndrome following endovascular intervention.

**Methods:**

For this meta-analysis, we conducted a systematic literature review of PubMed, Cochrane Library, and Embase databases from inception to April 14, 2021 for studies on ET for SVC syndrome. Studies included full-length journal articles on the use of ET among adults with SVC syndrome. Case reports or case series with fewer than 20 patients were excluded. We evaluated the endpoints of technical success rate, restenosis rate, and recurrence rates in SVC syndrome patients after endovascular stenting. The results of this study were calculated using random-effects models.

**Findings:**

We identified 6,012 reports, of which 39 studies met our inclusion criteria and were included for analysis. A total of 2200 patients received ET for SVC syndrome. The weighted technical success rate was 98.8% (95% CI 98.2–99.3) with low heterogeneity (I^2^=17.4%, *p* = 0.185), restenosis rate was 10.5% (95% CI 8.4–12.6) with moderate heterogeneity (I^2^=53.5%, *p*<0.001), and recurrence rate was 10.8% (95% CI 8.1–13.5) with high heterogeneity (I^2^=75.8%, *p*<0.001). Total complication rate was 8.6% (95% CI 7.3%-9.9%) with a mean complication rate of 7.5% (95% CI 4.7%-10.3%).

**Interpretation:**

Our systematic review revealed high technical success, low restenosis, and low recurrence rates following ET. Collectively, these results support the paradigm of ET as an effective and safe treatment for patients with SVC syndrome.

**Funding:**

None.

**Research in context**

**Evidence before this study**

The use of endovascular therapy (ET) in SVC syndrome has not previously been examined in a formal systematic review. Literature search for this meta-analysis was performed using PubMed (NLM), Embase (Elsevier) and Cochrane Central (Wiley) from inception to April 14,2021 with no language restrictions. Only full-length journal articles examining ET for SVC syndrome in adults were included for analysis, and studies with fewer than 20 subjects were excluded.

**Added value of this study**

This systematic review assesses the cumulative contemporary literature on managing SVC syndrome with ET, and is the first to examine this mounting collection of evidence in a formal meta-analysis. Our findings highlight a high technical success rate, low restenosis and recurrence rates.

**Implications of all the available evidence**

The current sum of literature demonstrates the safety and efficacy of ET for SVC syndrome. Our study demonstrates the continued need for large cohort and randomized controlled trials examining the use of ET in SVC syndrome. Further studies may also benefit from examining the impact of stent type on short and long-term outcomes, as well as the utility of concurrent catheter-directed thrombolysis.

Alt-text: Unlabelled box

## 1 Introduction

Superior vena cava (SVC) syndrome refers to the constellation of clinical manifestations caused by obstruction of venous flow due to external compression or internal stenosis or occlusion of the SVC. SVC syndrome affects ~15,000 patients in the United States annually [1]. Malignancies such as primary lung cancer are the most common cause accounting for 70% of cases but the recently increased utilization of indwelling intravascular devices such as catheters, and pacemaker/defibrillator leads have led to a rise in device-related SVC syndrome [2], [3], [4]. Consensus guidelines for SVC syndrome are lacking, however, traditionally treatment approach has included radiation therapy (RT) with or without chemotherapy, surgical bypass, or endovascular therapy (ET) [5].

In contemporary practice, compared to RT or surgical alternatives, ET has become the first-line treatment for the majority of patients with malignancy-related SVC. Although there are no randomized studies regarding ET in SVC syndrome, observational data have shown rapid relief of symptoms, high technical success rate, and low procedural complications [6], [7], [8], [9], [10], [11]. Optimal treatment of device-related SVC syndrome is not well defined, but ET remains a viable first option as it does not preclude or affect the outcome of potential open surgical bypass in the future [12]. As more patients undergo treatment with indwelling catheters, and potentially longer dwell times, the incidence of SVC syndrome is expected to increase [13].

As of yet, no formal systematic review or meta-analysis of all available literature regarding ET in treatment of SVC syndrome has been published. To address this knowledge gap, we performed a systematic review to assess the contemporary outcomes of SVC syndrome following ET.

## 2 Methods

### 2.1 Search strategy and selection criteria

For this quantitative meta-analysis of full-length journal articles on the use of ET for the treatment of SVC syndrome, we performed a systematic literature review. To identify studies to include or consider for this systematic review, the review team worked with a medical librarian (SCR) to develop detailed search strategies for each database. The search was developed for PubMed (NLM) and was translated to Embase (Elsevier) and Cochrane Central (Wiley) using a combination of keywords and subject headings. A gray literature search included ClinicalTrials.gov and the TRIP database. The search included no major limits and was limited to 1988 to present studies. The final search was completed on September 25, 2020. The search was updated on April 14, 2021.

PubMed (NLM) *from 1988 to 9/25/2020* (3593 Results)

PubMed (NLM) *from 9/25/2020 to* 4/14/2021 (136 Results)

Embase (Elsevier) *from 1988 to 9/25/2020* (3333 Results)

Embase (Elsevier) *from 9/25/2020 to* 4/14/2021 (187 Results)

Cochrane Central (Wiley) *from 1988 to 9/25/2020* (81 Results)

Cochrane Central (Wiley) *from 9/25/2020 to* 4/14/2021 (7 Results)

### 2.2 Study screening and data extraction

**Fig. 1:**
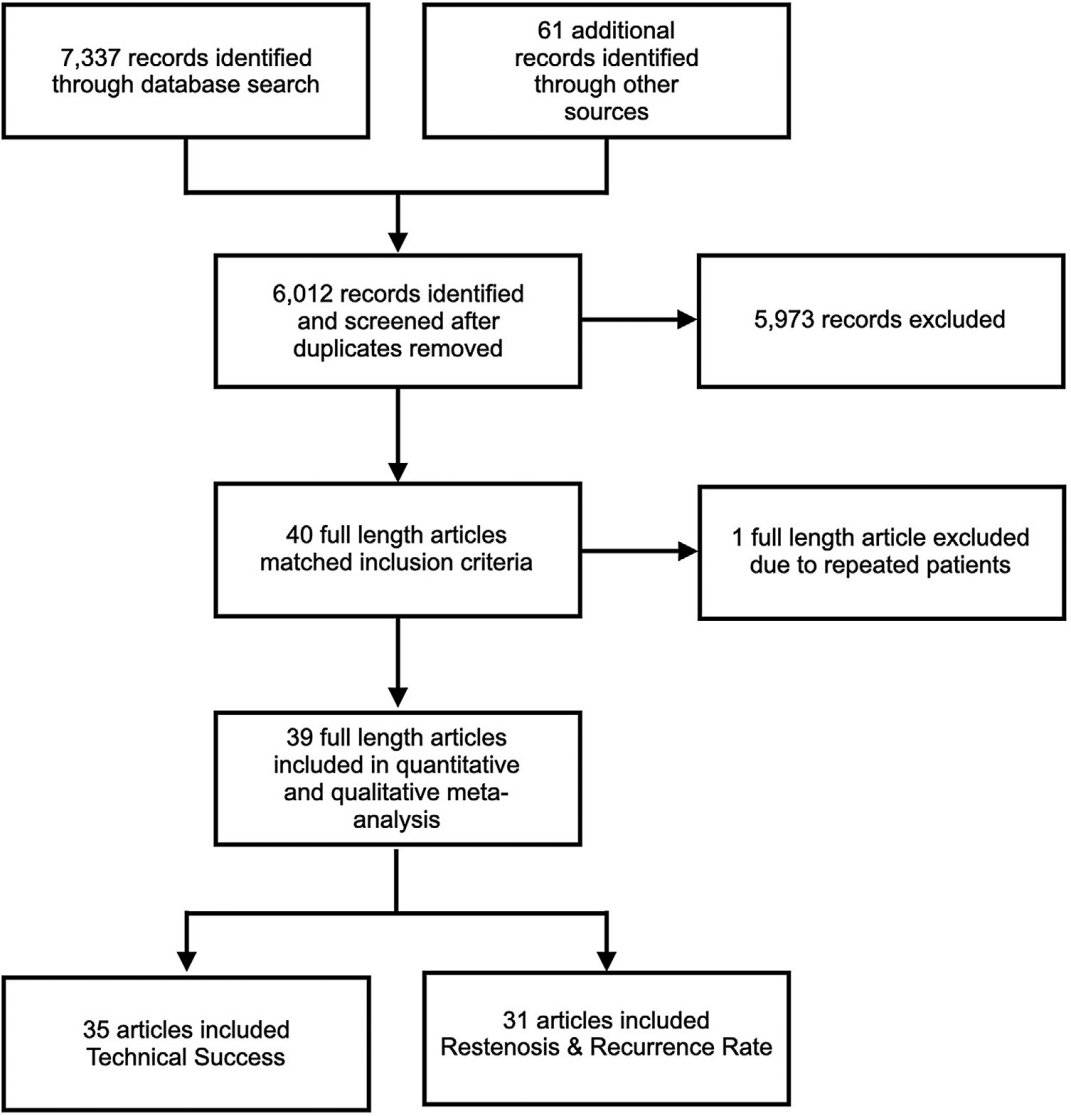
Flow diagram of study selection.

The search resulted in 7398 studies (61 from gray literature sources). 1386 duplicate studies were found and omitted using Endnote X.7 for the deduplication of records and 6012 references were eligible to screen. Studies were screened by title and abstract using Rayyan QCRI software by two blinded and independent reviewers (AHA and MZ). If a tiebreaker was needed, a third reviewer was called in to decide (IS). This process was repeated for full-text article screening and article selection. Studies were included if they were full-length journal articles describing original research on SVC syndrome and ET among adult human (>18 years) subjects. Studies were excluded if they were case reports, or case series with < 20 patients to allow sufficient sample size, reject the null hypothesis, and to minimize the influence of isolated reports of unusual phenomena (Fig. 1). The full search details and PICOTS elements of inclusion and exclusion are provided in Appendix 1 and 2.

### 2.3 Outcomes

Primary outcomes included technical success rate, restenosis rate, and SVC syndrome recurrence rate. Secondary outcomes included primary patency, and secondary patency. These results and associated 95% CIs were used with a random-effects model for primary analyses. Subgroup analysis of primary patency, and secondary patency in group of patients with malignant SVC syndrome (MSVC) and benign SVC syndrome (BSVC) was also performed (Appendix 3).

### 2.4 Quality assessment

**Fig. 2:**
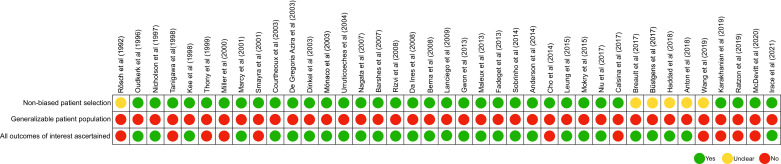
Quality assessment for included studies. The quality of all included studies was assessed using methodology described by Kansagara et al. [14].

The quality of all included studies was assessed using methodology described by Kansagara et al. [14]. Quality Assessment was performed by two independent reviewers (AHA and MZ) with any discrepancies resolved by a third investigator (IS). Studies were examined for the use of non-biased patient selection, overall generalizability, as well as whether results for their desired outcome measures were attained (Fig. 2).

### 2.5 Statistical analysis

Results of studies investigating SVC Syndrome and ET in the general population were obtained from individual studies using a Microsoft Excel spreadsheet. Data analysis was performed using OpenMeta [Analyst] [15] which uses metafor [16] to calculate weighted estimates. Metafor uses the Freeman-Tukey double arcsine transformed proportion [17] for variance stabilizing transformation. Proportions and 95% confidence intervals (CIs) were calculated using binary random-effects models. Each study was weighted by inverse of its variance. Heterogeneity among studies was assessed using Cochran Q and I^2^ statistics. To assess for presence of publication bias, we used funnel plots and Egger test for each end point (Appendix 5).

### 2.6 Role of the funding source

There was no funding for this study. The corresponding authors had full access to all of the data and assumed final responsibility to submit for publication.

## 3 Results

Our search strategy yielded 7398 potential reports, and 6012 records were identified for eligibility screening after duplicates were removed. Based on our selection criteria, 39 full-length original research articles were included for analysis. One study was excluded based on patients being re-studied in a subsequently more recent publication [18]. Quality of included studies were assessed as described. As expected, the individual studies included in this meta-analysis had overall poor generalizability to the general population. Most of the studies were primarily single-center retrospective studies with one prospective study. All studies reported their desired outcomes of interest. An overview of study characteristics is detailed in Table 1. Publication dates for eligible studies ranged from 1992 to 2021 and study sizes ranged from 20 to 183, with a total of 2200 patients.

**Table 1:** Study characteristics.

	*N*	Stents Used	Age [Mean or Median^1^ (Range)]	Bilateral Stents Used? [n (%)]	Complications [n (%)]	Primary Patency%	Secondary Patency%	Technical Success%	Clinical Success%
Rösch et al. [37] (1992)	22	Gianturco	56^†^ (28–68)	0 (0.0)	1 (4.6)	NA	NA	100	100
Oudkerk et al. [38] (1996)	30	Wallstent; Z-stent	60 (40–74)	0 (0.0)	NA	NA	NA	100	86.7
Nicholson et al. [26] (1997)	76	Wallstent	60.4 (45–78)	8 (10.5)	8 (10.5)	NA	NA	100	NA
Tanigawa et al. [39] (1998)	23	Gianturco	61.2 (35–79)	3 (13.0)	NA	NA	NA	100	78.3
Kee et al. [24] (1998)	59	Palmaz; Wallstent; Gianturco Z-stent	52.8 (28–83)	1 (1.7)	6 (10.2)	78.6, (MSVC) 76.9 (BSVC)	92.9, (MSVC) 84.6, (BSVC)	94.9	NA
Thony et al. [40] (1999)	26	Wallstent; Strecker	54 (26–81)	0 (0.0)	1 (3.9)	83.0	89.0	NA	92.3
Miller et al. [41] (2000)	23	Wallstent	64 (26–89)	0 (0.0)	0 (0.0)	NA	NA	100	82.6
Marcy et al. ^32^(2001)	39	Gianturco; Strecker; Memotherm	59 (17–79)	0 (0.0)	NA	92.3	NA	97.4	92
Smayra et al. [25](2001)	30	Memotherm; Wallstent; Symphony	61 (29–86)	NA^2^	2 (6.7)	74 (MSVC) 50 (BSVC) 22 (HD AV Fistula)	74 (MSVC) 75 (BSVC) 56 (HD AV Fistula)	NA	NA
Courtheoux et al. [42] (2003)	20	Wallstent	58 (35–74)	0 (0.0)	0 (0.0)	94.0	NA	100	94.7
De Gregoria Azira et al. ^7^(2003)	82	Wallstent; Palmaz	57.8 (39–79)	0 (0.0)	0 (0.0)	92.6 (MSVC) 57.1 (BSVC)	98.5 (MSVC) 100 (BSVC)	100	95.1
Dinkel et al. [20](2003)	84	Wallstent	64 (39–79)	61 (72.6)	20 (23.8)	90 (1 month) 81 (3 months) 76 (6 months) 69 (12 months) 61 (24 months)	NA	98.8	90.4
Mónaco et al. [43] (2003)	44	Wallstent	55.6 (21–77)	0 (0.0)	0 (0.0)	NA	NA	100	90.9
Urruticoechea et al. [44] (2004)	52	Wallstent; Memotherm	57 (NA)	0 (0.0)	10 (19.2)	NA	NA	100	53
Nagata et al. [8] (2007)	71	Wallstent; S-Z stent; M-Z stent; O-Z stent	63.4 (30–85)	3 (4.2)	11 (15.5)	87.7	95.4	100	87.3
Barshes et al. [11] (2007)	56	Wallstent; Palmaz; Johnson & Johnson Interventional System; Warren	62.6 (32–82)	9 (16.1)	0 (0.0)	64 (MSVC) 76 (BSVC)	NA	100	96.4
Rizvi et al. [12] (2008)	32	Wallstent; SMART; Palmaz; Viabahn; Lumminexx; Protégé	41 (5–75)	0 (0.0)	1 (3.1)	96.0	96.0	87.5	93
Da Ines et al. [45] (2008)	34	Wallstent	60.5 (44–81)	0 (0.0)	2 (5.9)	81.0	100	100	100
Berna et al. [46] (2008)	31	NA	55.6 (39–76)	0 (0.0)	1 (3.2)	100 (6 months) 93 (6 months)	NA	100	100
Lanciego et al. [47] (2009)	149	Wallstent	65 (44–84)	12 (8.1)	30 (20.1)	86.6	93.3	100	82.6
Gwon et al. [36] (2013)	73	Covered ePTFE vs uncovered	60.3 (35–81)	0 (0.0)	0 (0.0)	NA	NA	100	93.2
Maleux et al. [48] (2013)	78	Zilver, Cook Medical	64.1 (35–85)	0 (0.0)	1 (1.3)	89	NA	98.7	99
Fadeget et al. [6] (2013)	164	Wallstent; Memotherm; SMART; Strecker; Protégé	59.9 (NA)	16 (9.8)	21 (12.8)	78.0	95.1	84.5	NA
Sobrinho et al. [19] (2014)	56	Sinus-XL; SMART; Wallstent; Express	59.3 (34–84)	0 (0.0)	9 (16.1)	92.0	NA	100	86
Andersen et al. [33] (2014)	25	E*Luminexx; Sinus-XL; Zilver Vena	65 (49–86)	0 (0.0)	0 (0.0)	NA	NA	100	96
Cho et al. [21] (2014)	40	ComVi	61.4 (35–81)	0 (0.0)	6 (15.0)	95 (1 month) 92 (3 months) 86 (6, 12 months)	NA	100	92
Leung et al. [49] (2015)	56	Wallstent	64 (48–83)	0 (0.0)	13 (23.2)	NA	NA	97.0	93
Mokry et al. [50] (2015)	23	Sinus XL	62.5 (51–83)	0 (0.0)	4 (17.4)	95.7	100	100	NA
Niu et al. [22] (2017)	47	Sinus XL; Zilver; Luminexx; Smart	NA	0 (0.0)	0 (0.0)	93.4 (3 months) 87.4 (6 months) 81.2 (12 months)	NA	100	100
Calsina et al. [51] (2017)	33	Wallstent; Protégé	57.6 (34–71)	0 (0.0)	3 (9.0)	94 (1, 3, 6, 12 months)	97 (1, 3, 6, 12 months)	100	85
Breault et al. [52] (2017)	44	Wallstent	56^†^ (5–88)	0 (0.0)	2 (4.6)	NA	NA	97.7	97.7
Büstgens et al. [23] (2017)	141	SMART stent; Wallstent; Zilverstent; Epic stent	64.6^†^ (36–84)	0 (0.0)	NA	94 (2 months) 83.7 (6 months) 85.7 (12, 24 months)	NA	NA	NA
Haddad et al. [53] (2018)	59	Wallstent; Protégé; Smart; Gore Biabahn; iCast	44 (24–71)	0 (0.0)	1 (1.7)	NA	NA	100	100
Anton et al. [54] (2018)	31	Sinus XL; OptiMed; Protégé; EverFlex; Covidien; Ireland	67 (NR)	0 (0.0)	NA	Sinus XL: 100 (3, 6, 12 months) Protégé: 84 (3, 6 months); 56 (12 months)	NA	100	100
Wang et al. [55] (2019)	64	Fluency (covered); Luminexx (uncovered)	61.6 (NR)	0 (0.0)	NA	89	NA	100	100
Karakhanian et al. [56] (2019)	28	Wallstent; Sinus; Sioxx	52.5 (37–68)	0 (0.0)	0 (0.0)	NA	NA	96.4	96.4
Ratzon et al. [34] (2019)	183	NA	59^†^ (NA)	0 (0.0)	0 (0.0)	NA	NA	NA	NA
McDevitt et al. [57] (2020)	30	Gianturco Z-Stent	48.6 (16–89)	0 (0.0)	NA	NA	NA	100	NA
Irace et al. [58] (2021)	42	Memotherm; Wallstent	72 (NA)	0 (0.0)	5 (11.9)	64	NR	100	NR

Restenosis Number [n (%)]	Recurrence Number [n (%)]	Thrombolysis Given?	Stanford Doty Criteria Used?	Recurrence Time to Follow Up (Maximum in Months)	Recurrence Time to Follow Up (Mean in Months)	Chronicity of Presentation (Acute or Subacute) [n (%)]	Chronicity of Presentation (Chronic) [n (%)]	Post Procedure Anticoagulation Regimen	
NA	1 (4.6)	Yes	No	16	NA	12 (54.5)	10 (45.4)	Coumadin	
3 (10.0)	3 (10.0)	Yes	No	34	2.5	30 (100)	0 (0.0)	Coumadin	
9 (11.8)	7 (9.2)	Yes	No	6	NA	NA	NA	Coumadin or Heparin	
NA	1 (4.4)	Yes	No	40	NA	NA	NA	Heparin	
5 (8.5)	2 (3.4)	Yes	No	34	7	27 (45.8)	32 (54.2)	Coumadin	
3 (11.5)	3 (11.5)	Yes	No	10	NA	4 (15.4)	22 (84.6)	Aspirin or Heparin or Coumadin	
NA	4 (17.4)	No	No	8	NA	18 (78.2)	5 (21.7)	Aspirin or Oral Anticoagulation	
1 (2.6)	3 (7.7)	Yes	No	24	6	NA	NA	Aspirin	
5 (16.7)	NA	Yes	Yes	NA	NA	2 (6.7)	28 (93.3)	NA	
3 (15.0)	3 (15.0)	No	No	NA	NA	NA	NA	LMWH followed by Coumadin and Aspirin	
7 (8.5)	1 (1.2)	Yes	No	61	11	82 (100)	0 (0.0)	LMWH	
19 (22.6)	8 (9.5)	No	No	55	13.9	NA	NA	Coumadin	
4 (9.1)	6 (13.6)	No	No	NA	NA	NA	NA	LMWH	
9 (17.3)	5 (9.6)	No	No	NA	NA	NA	NA	LMWH or Heparin or Coumadin	
8 (11.3)	8 (11.3)	No	No	29	NA	NA	NA	Coumadin or Antiplatelet	
4 (9.1)	3 (5.4)	Yes	No	57	14	2 (3.6)	3 (96.4)	Coumadin	
1 (3.1)	1 (3.1)	Yes	Yes	77	26	2 (6.2)	30 (93.8)	Coumadin	
3 (8.8)	5 (14.7)	Yes	No	NA	NA	4 (11.8)	30 (88.2)	NA	
2 (6.5)	0 (0.0)	Yes	No	12	NA	NA	NA	Oral Anticoagulation	
20 (13.4)	20 (13.4)	No	Yes	NA	5	NA	NA	Coumadin	
12 (16.4)	12 (16.4)	No	No	28	NA	NA	NA	Coumadin or Aspirin	
8 (10.3)	8 (10.3)	Yes	No	NA	NA	5 (6.4)	73 (93.6)	LMWH and Aspirin	
16 (9.8)	36 (22.0)	Yes	No	NA	NA	30 (18.3)	134 (81.7)	Coumadin	
2 (3.6)	6 (10.7)	No	No	NA	4	NA	NA	LMWH and Aspirin	
2 (8.0)	3 (12.0)	No	No	16	NA	NA	NA	Aspirin	
NA	NA	No	No	NA	NA	NA	NA	Oral Anticoagulation	
8 (14.3)	9 (16.1)	NA	No	NA	2	NA	NA	Oral Anticoagulation	
1 (4.4)	1 (4.4)	Yes	Yes	10	6	NA	NA	Heparin	
6 (12.8)	6 (12.8)	Yes	No	13	NA	1 (2.1)	46 (97.9)	Coumadin	
2 (6.1)	NA	NA	No	NA	NA	NA	NA	Antiplatelet or Oral Anticoagulation	
13 (29.6)	9 (20.5)	No	Yes	190	42	NA	NA	Oral Anticoagulation	
NA	NA	NA	No	NA	NA	NA	NA	Variable	
20 (33.9)	23 (39.0)	No	Yes	133	24	NA	NA	Coumadin or LMWH	
3 (9.7)	2 (6.5)	No	Yes	NA	6	NA	NA	Oral Anticoagulation or Antiplatelet	
7 (10.9)	NA	No	No	NA	NA	NA	NA	Coumadin	
NA	NA	No	Yes	NA	NA	NA	NA	Dual Antiplatelet or Oral Anticoagulation	
NA	NA	No	No	NA	NA	NA	NA	Oral Anticoagulation or None	
NA	NA	NA	No	NA	NA	NA	NA	Dual Antiplatelet and Coumadin	
3	13	Yes	Yes	24	24	42 (100.0)	0 (0.0)	LMWH followed by Oral Anticoagulation and Aspirin	

1 † denotes median value.

2 Bilateral stenting was performed, however the number of patients receiving bilateral stents was not reported. Abbreviations: NA Not Applicable, MSVC Malignant Superior Vena Cava Syndrome, BSVC Benign Superior Vena Cava Syndrome, HD Hemodialysis, AV Arteriovenous, LMWH Low Molecular Weight Heparin.

### 3.1 Technical success

**Fig. 3:**
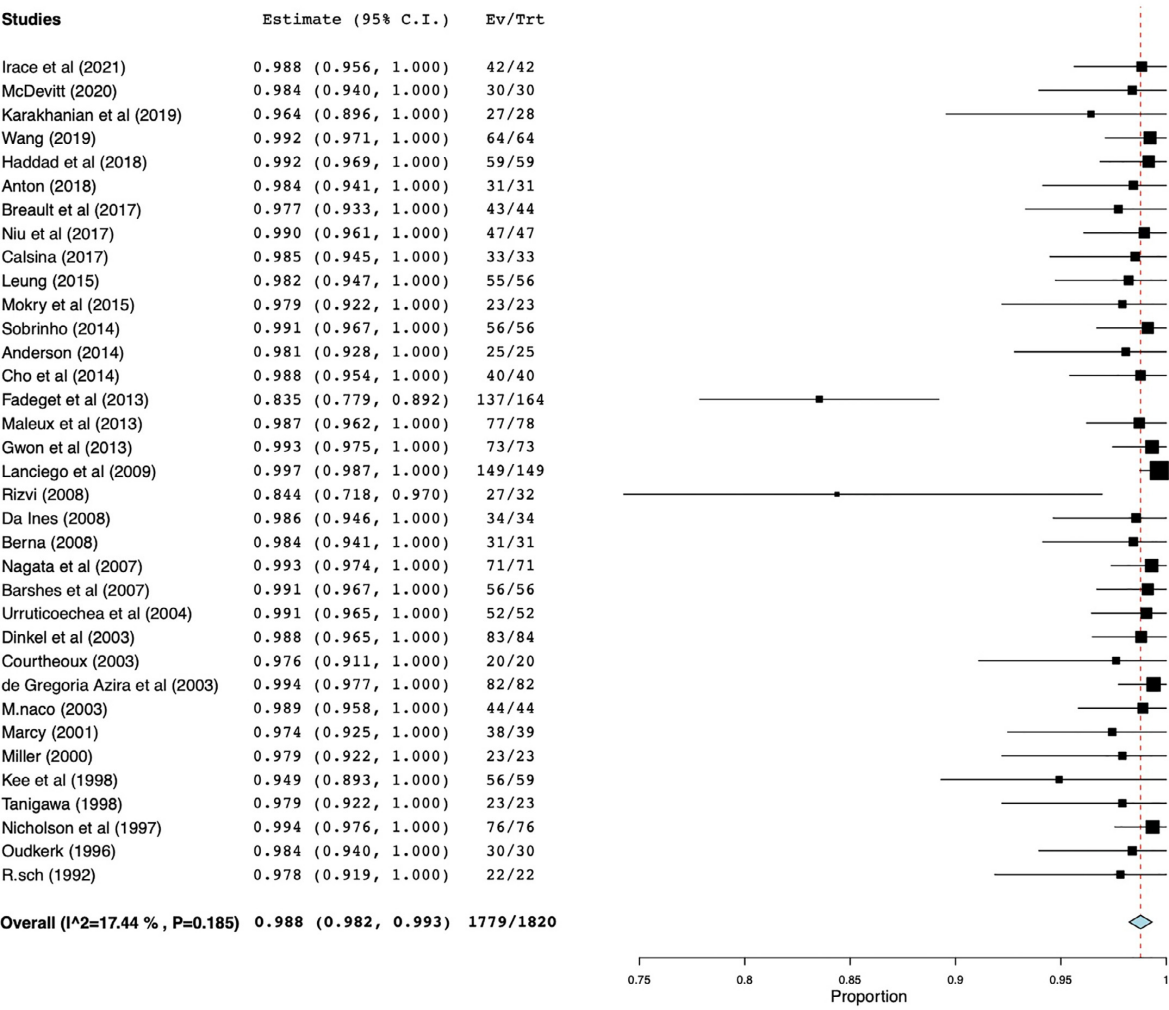
Forest plot for technical success. Technical success was defined as angiographic evidence of vessel stenosis resolution post stent deployment and reported in 35 individual studies with a total of 1820 patients.

Technical success was defined as angiographic evidence of vessel stenosis resolution post stent deployment and reported in 35 individual studies with a total of 1820 patients. Quantitative synthesis showed that the weighted technical success rate was 98.8% (95% CI 98.2%–99.3%) with a range of 83%–100% (Fig. 3). 26 out of 35 studies reported a technical success rate of 100% (Table 1). The number of stents required to achieve technical success was variable and not always reported. One study with a total number of 149 patients reported technical success with one stent for 102 patients (68.5%), two stents for 36 patients (24.2%), three stents for ten patients (6.7%), and four stents for one patient (0.7%) [5]. In general, nine studies reported use of bilateral stents although one study did not report the number of bilateral stents used. 113 total patients received bilateral stents among the eight studies and the mean percentage of bilateral stents used was 17% with a range of 1.7 to 72.6%.

### 3.2 Restenosis and recurrence rate

**Fig. 4:**
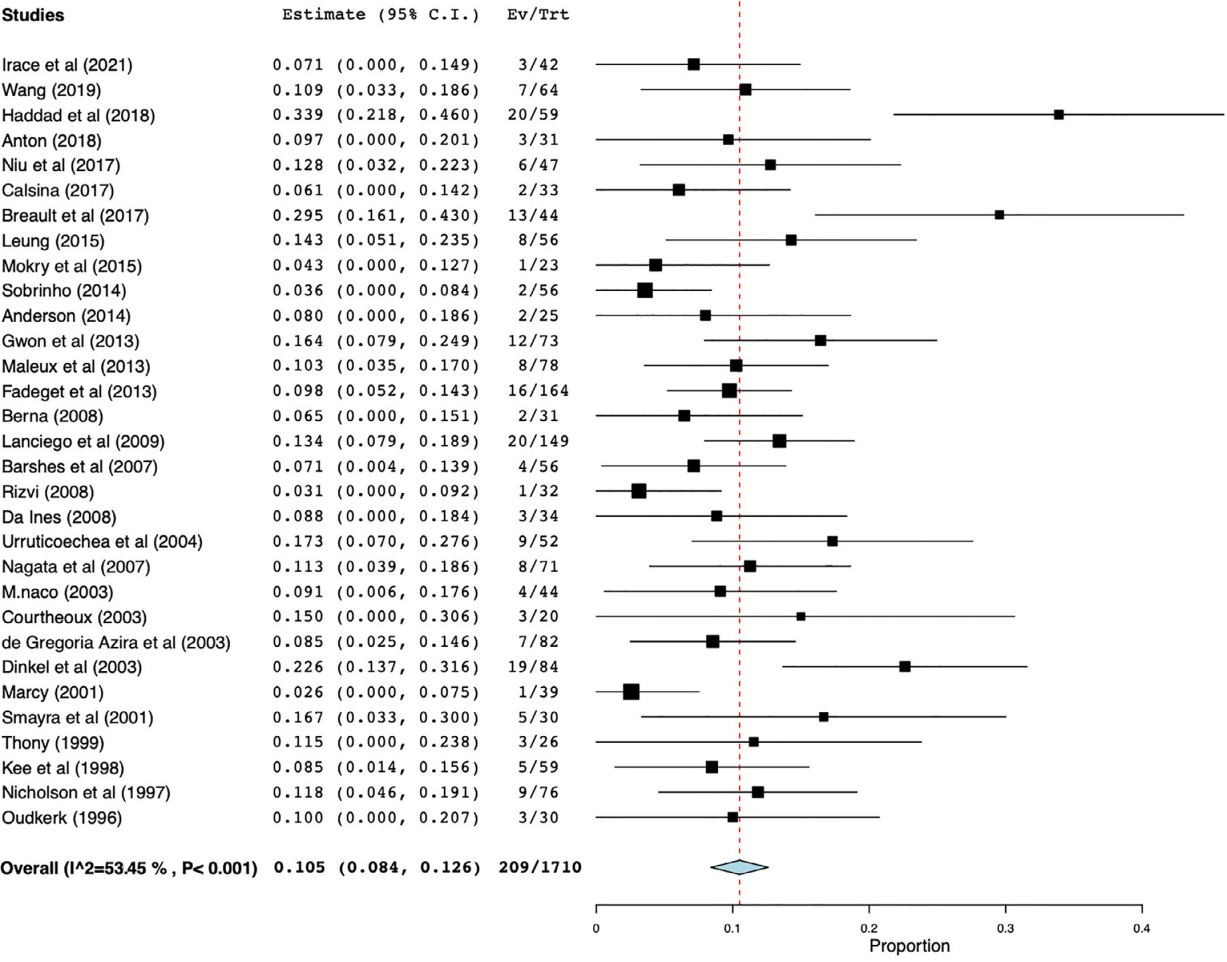
Forest plot for restenosis. Restenosis was defined as obstruction of the endoprosthesis by occlusion or stent thrombosis and was reported in 31 studies with a total of 1710 patients.

Restenosis was defined as obstruction of the endoprosthesis via occlusion or stent thrombosis and was reported in 31 studies with a total of 1710 patients. Weighted restenosis rate was 10.5% (95% CI 8.4%–12.6%), range of 2.6%–34% with moderate heterogeneity (I^2^=53.5%, *p* < 0.001). (Fig. 4). Stent migration, stent shortening, and incorrect stent placement were some of the reported causes of early stent restenosis or occlusion [5,6,8,19].

**Fig. 5:**
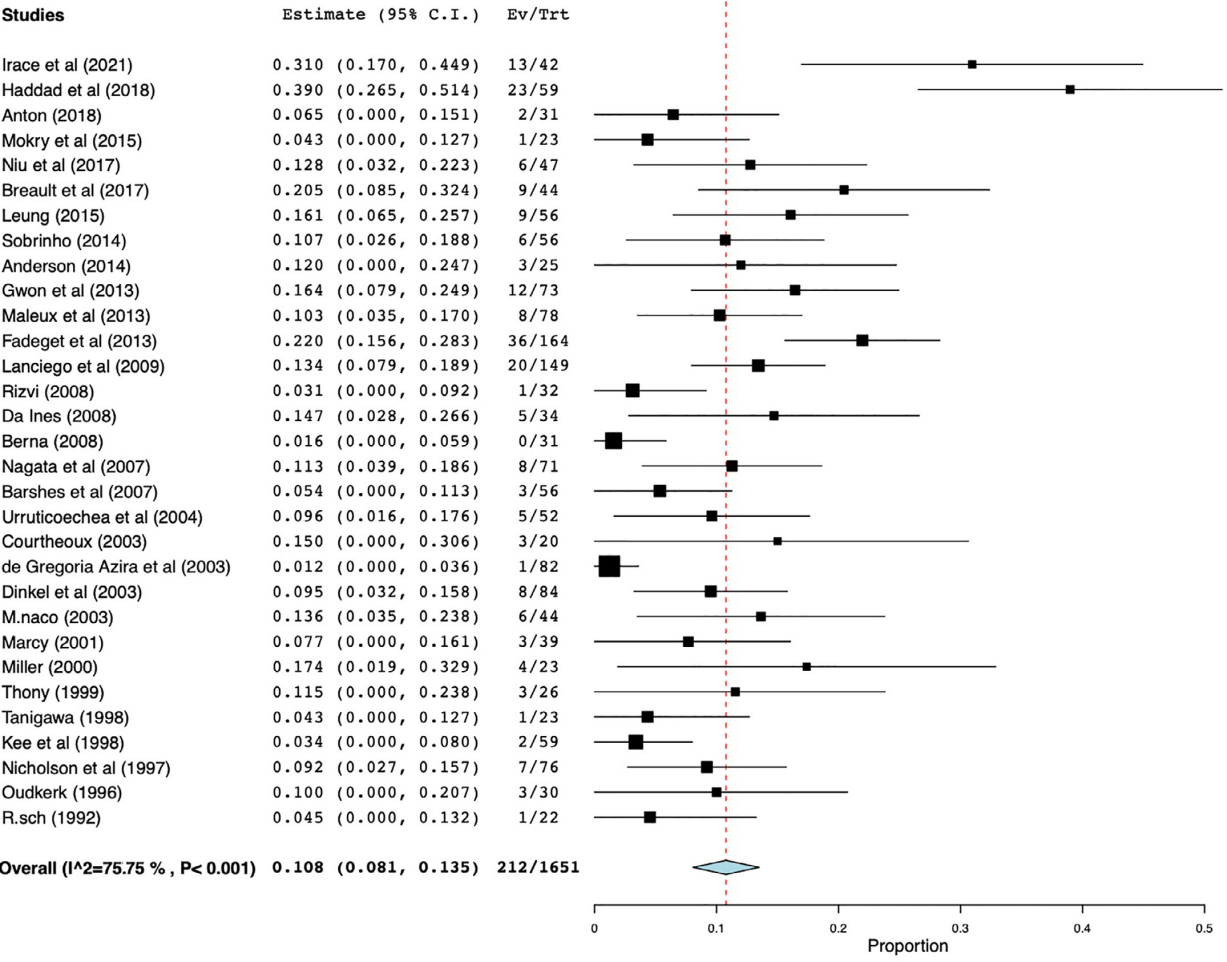
Forest plot for SVC syndrome recurrence. Recurrence was defined as the recurrence of SVC syndrome symptoms after stent therapy and was also reported in 31 studies with a total of 1651 patients.

Recurrence was defined as the re-emergence of SVC syndrome symptoms after stent therapy and was also reported in 31 studies with a total of 1651 patients. Weighted recurrence rate of SVC syndrome was 10.8% (95% CI 8.1%–13.5%) ranging from 1.6%–39% with high heterogeneity (I^2^=75.8%, *p* < 0.001) (Fig. 5). 21 studies reported maximum follow-up intervals with a mean of 41.8 (SD=45.2) months and range of six to 190 months. 15 studies reported the average follow-up interval with an overall mean of 12.9 (SD=11.4) months and a range of two to 42 months.

### 3.3 Secondary end points

**Fig. 6:**
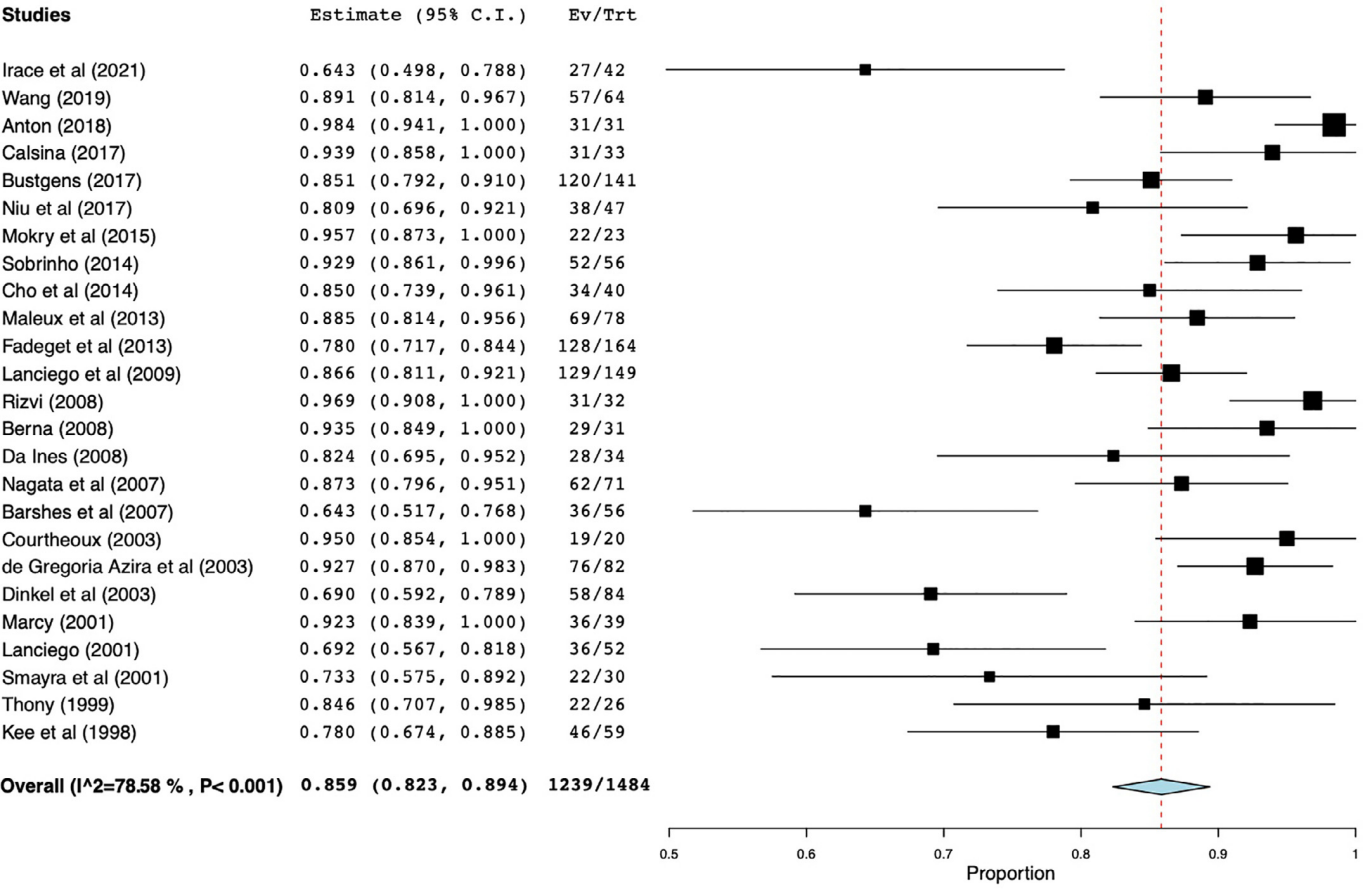
Forest plot for primary patency. Primary patency rate was defined as the percentage of stents that remained patent after 12 months in patients with all etiologies of SVC syndrome (MSVC) and was reported in 24 studies with a total of 1484 patients.

Primary patency rate was defined as the percentage of stents that remained patent after 12 months in patients with all etiologies of SVC syndrome (MSVC) and was reported in 24 studies with a total of 1484 patients (Fig. 6). Weighted primary patency rates were reported to be 85.9% (95% CI 82.3%–89.4%) ranging from 64%–98% with high heterogeneity (I^2^ = 78.6%, *p* < 0.001). Four studies reported primary patency rates at several intervals with rates predictably decreasing with increasing intervals [20], [21], [22], [23]. For example, Dinkel et al. [20] reported primary patency rates at 1, 3, 6, and 12 months to be 90%, 81%, 76%, and 69%, respectively [7,11,24,25].

**Fig. 7:**
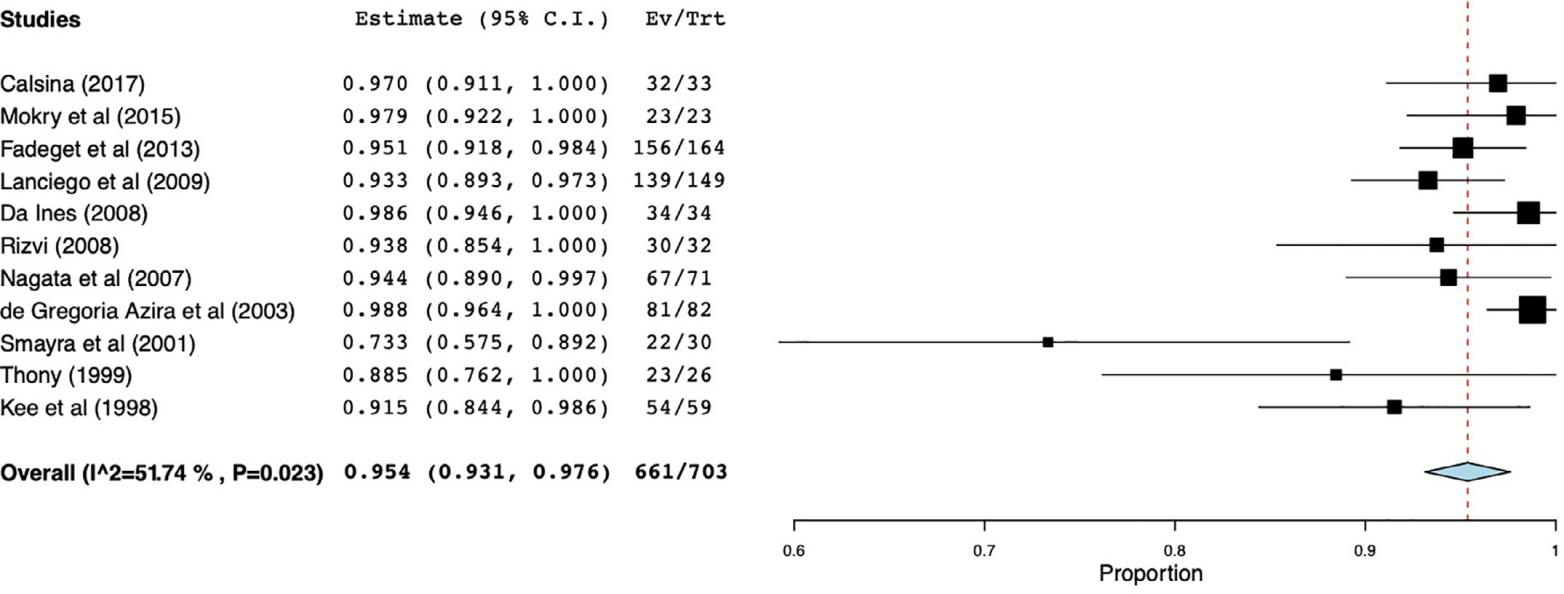
Forest plot for secondary patency. Secondary patency rates in patients with all etiologies of SVC syndrome over a 12-month period were discussed in only 11 studies with a total of 703 patients.

Secondary patency rates in patients with all etiologies of SVC syndrome over a 12-month period were discussed in only 11 studies with a total of 703 patients (Fig. 7). Weighted secondary patency rate was reported to be 95.4% (95% CI 93.1%–97.6%) ranging from 73.3%–98.8% with moderate heterogeneity (I^2^ = 51.7%, *p* = 0.023).

Sensitivity analysis of primary patency of MSVC syndrome was reported to be 86.0% (95% CI 82.4%–89.7%) ranging from 64%–98% with high heterogeneity (I^2^ = 75.6%, *p* < 0.001). Primary patency of BSVC syndrome was reported to be 75.8% (95% CI 58.2%–93.5%) ranging from 57%–97% with high heterogeneity (I^2^ = 78.9%, *p* < .001). Of note, MSVC syndrome cohort was 1957 but only 1301 reported primary patency. Of the BSVC syndrome cohort of 197 patients, only 92 reported primary patency. Similarly, secondary patency of MSVC syndrome was reported to be 96.1% (95% CI 94.2%–97.9%) ranging from 79%–99% with low heterogeneity (I^2^ = 29.8%, *p* = 0.171). Secondary patency of BSVC syndrome was reported to be 86.3% (95% CI 72.5%–100.0%) with high heterogeneity (I^2^ = 82.1%, *p* < 0.001).

To further assess the high heterogeneity, mean/median age of the participants in each included study was used to perform a random-effects meta-regression analysis (Appendix 4), and it did not reveal any significant association with the individual end-points (restenosis, recurrence, primary patency, and secondary patency) to explain the high heterogeneity. Similarly, duration of follow-up was evaluated in a random-effects model for meta-regression analysis which revealed significant association with the heterogeneity with the restenosis outcome, but not with the other end-points evaluated. Publication bias was noted for all outcomes based on funnel plots and by Egger test (*P*-value < 0.05 for all outcomes) (Appendix 5).

Complication rates were reported in 32 studies (1843 patients) with a total complication rate of 8.6% (95% CI 7.3%–9.9%) and mean complication rate of 7.5% (95% CI 4.7%–10.3%); ten studies reported zero complications (Table 1). Minor complications were reported at 1.1% and included local pain, hematoma, and local infection at the puncture site. Major complications were reported at 3.7% and included in-stent restenosis and obstruction (*n* = 24), thrombosis (*n* = 16), stent migration (*n* = 14), cardiac tamponade (*n* = 5), acute pulmonary edema (*n* = 6) and respiratory distress (*n* = 3). Complications resulting in death, however, was exceedingly rare and reported in only 12 patients (0.7%), mostly due to cardiac tamponade, acute pulmonary embolism, and respiratory insufficiency (Table 2).

**Table 2:** Complication types.

	Total [n (%)]	Rösch et al. (1992)	Nicholson et al. (1997)	Kee et al. (1998)	Thony et al. (1999)	Smayra et al. (2001)	Dinkel et al. (2003)	Urruticoechea et al. (2004)	Nagata et al. (2007)	Rizvi et al. (2008)	Da Ines et al. (2008)
Obstruction or occlusion	24 (15.3)	–	–	–	–	–	8	–	–	–	–
Thrombosis	17 (10.8)	1	–	–	–	–	–	–	–	–	–
Stent migration	14 (8.9)	–	–	1	–	1	–	1	3	–	–
Late recurrence	11 (7.0)	–	–	–	–	–	11	–	–	–	–
Hematoma	11 (7.0)	–	1	1	–	–	–	–	–	1	–
Bleeding (anticoagulation-related)	7 (4.5)	–	–	–	–	–	–	6*	–	–	–
Local pain	7 (4.5)	–	–	–	–	–	–	–	7	–	–
Arrythmia	6 (3.8)	–	–	1	–	–	–	1	–	–	–
Stent shortenings	6 (3.8)	–	–	–	–	–	–	–	–	–	–
Acute pulmonary edema	6 (3.8)	–	–	–	–	–	–	–	–	–	–
Fever	6 (3.8)	–	–	–	–	–	–	–	–	–	–
Pulmonary embolism	6 (3.8)	–	2	1*	–	–	–	–	1	–	–
Hemoptysis	5 (3.2)	–	–	–	–	–	–	–	–	–	–
Cardiac tamponade	5 (3.2)	–	–	–	–	1*	1*	–	–	–	–
Persistent arm swelling	3 (1.9)	–	3	–	–	–	–	–	–	–	–
Acute respiratory distress	3 (1.9)	–	–	1*	–	–	–	–	–	–	2
Sepsis	2 (1.3)	–	–	–	–	–	–	2	–	–	–
Incorrect stent placement	2 (1.3)	–	–	–	–	–	–	–	–	–	–
Epistaxis	2 (1.3)	–	–	–	–	–	–	–	–	–	–
Superficial wound infection	2 (1.3)	–	–	–	–	–	–	–	–	–	–
Deep vein thrombosis	1 (0.6)	–	1	–	–	–	–	–	–	–	–
Stent-related shoulder pain	1 (0.6)	–	1	–	–	–	–	–	–	–	–
Gastrointestinal hemorrhage (thrombolysis-related)	1 (0.6)	–	–	1	–	–	–	–	–	–	–
Insufficient stent expansion	1 (0.6)	–	–	–	–	–	–	–	–	–	–
Axial stent plication	1 (0.6)	–	–	–	–	–	–	–	–	–	–
Rectal bleeding	1 (0.6)	–	–	–	–	–	–	–	–	–	–
Hoarseness of voice	1 (0.6)	–	–	–	–	–	–	–	–	–	–
Impaired venous drainage of upper extremity	1 (0.6)	–	–	–	–	–	–	–	–	–	–
Pericardial effusion	1 (0.6)	–	–	–	–	–	–	–	–	–	–
Fibrinolysis	1 (0.6)	–	–	–	1*	–	–	–	–	–	–
Intracranial hemorrhage	1 (0.6)	–	–	–	–	–	–	–	–	–	–
Other	2 (1.3)	–	–	–	–	–	–	–	–	–	–

Berna et al. (2008)	Lanciego et al. (2009)	Maleux et al. (2013)	Fadeget et al. (2013)	Sobrinho et al. (2014)	Cho et al. (2014)	Leung et al. (2015)	Mokry et al. (2015)	Calsina et al. (2017)	Breault et al. (2017)	Haddad et al. (2018)	Irace et al. (2021)
–	16	–	–	–	–	–	–	–	–	–	–
–	4	–	–	–	–	8	1	2	–	–	1*
–	1	1	2	3	–	–	1	–	–	–	–
–	–	–			–	–	–	–	–	–	–
–	–	–	3	1	–	2	–	–	–	–	2
–	–	–	–	–	–	1	–	–	–	–	–
–	–	–	–	–	–	–	–	–	–	–	–
–	–	–	–	3	–	–	–	1	–	–	–
–	6	–	–	–	–	–	–	–	–	–	–
–	–	–	4	–	–	1	–	–	1	–	–
–	–	–	1	–	5	–	–	–	–	–	–
1	–	–	–	–	–	–	1*	–	–	–	–
–	–	–	4*	1	–	–	–	–	–	–	–
–	–	–	–	1	–	1*	–	–	1*	–	–
–	–	–	–	–	–	–	–	–	–	–	–
–	–	–	–	–	–	–	–	–	–	–	–
–	–	–	–	–	–	–	–	–	–	–	–
–	2	–	–	–	–	–	–	–	–	–	–
–	–	–	2	–	–	–	–		–	–	–
–	–	–	–	–	–	–	–	–	–	–	2
–	–	–	–	–	–	–	–	–	–	–	–
–	–	–	–	–	–	–	–	–	–	–	–
–	–	–	–	–	–	–	–	–	–	–	–
–	1	–	–	–	–	–	–	–	–	–	–
–	–	–	1	–	–	–	–	–	–	–	–
–	–	–	1	–	–	–	–	–	–	–	–
–	–	–	–	–	1	–	–	–	–	–	–
–	–	–	–	–	–	–	1	–	–	–	–
–	–	–	–	–	–	–	–	–	–	1	–
–	–	–	–	–	–	–	–	–	–	–	–
–	–	–	1*	–	–	–	–	–	–	–	–
–	–	–	2	–	–	–	–	–	–	–	–

⁎ Indicates complication resulting in death of one patient Total complication rate was 8.6% (95% CI 7.3%−9.9%) with a mean complication rate of 7.5% (95% CI 4.7%−10.3%).

Clinical success was defined as a complete or partial resolution of symptoms of SVC syndrome including upper extremity, head, and neck edema, relief of facial discomfort and headache. Clinical success was reported in 32 studies and ranged from 53%–100% with an average of 91.7%. One study compared clinical success in a cohort of patients that received stent therapy versus RT for malignant SVC obstruction and reported clinical success of 96% in the stent group and 56% in the RT cohort [26]. A variety of stents were used in these studies with the most common being Wallstent (Boston Scientific, Natick, Massachusetts), Palmaz stents (Johnson & Johnson Interventional Systems, Warren, NJ), and Gianturco *Z*-stent (Cook Medical, Bloomington, Indiana).

All studies reported use of intraprocedural and post-procedural anticoagulation, but duration and outcomes were not reported. Studies reported use of either oral anticoagulants such as coumadin, anti-platelet agents (Aspirin, Clopidogrel), or parenteral anticoagulants (unfractionated heparin or low-molecular weight heparin). Several studies reported variable anticoagulation regimen based on underlying etiology of SVC syndrome. Duration ranged from 3 months to lifelong based on comprehensive risk assessment of each individual patient (Table 1).

## 4 Discussion

The treatment approach in patients with SVC syndrome is multidisciplinary and treatment options include radiation therapy (RT) with or without chemotherapy, surgical bypass, or ET such as angioplasty, stenting, and catheter-based thrombus removal. Traditionally, RT was viewed as the first-line treatment to relieve obstruction in patients with life-threatening symptoms due to SVC syndrome. RT has recently been less frequently used given the efficacy of endovascular stents, complications associated with high dose RT, delayed symptomatic relief, and obscuring of histological diagnosis after RT [27]. In a cohort of patients with malignant SVC obstruction treated with stent therapy versus RT, percutaneous stent placement was reported to be associated with immediate symptomatic relief, higher clinical success rates, and lower complication rates. Interestingly, 25% of patients in the RT cohort experienced initial worsening of symptoms attributed to radiation-induced edema [26].

The findings of our systematic review lend credence to ET as first-line treatment for SVC syndrome as it provides a rapid resolution of symptoms, high technical success, low restenosis and recurrence rates, with low intra- and post-procedural complications. Generally, SVC obstruction is categorized into four types using the Stanford and Doty Classification system based on major venographic patterns with each type associated with progressively advanced obstruction and development of collateral venous systems [4]. Nine studies reported using the Stanford and Doty Classification system (Table 1), but the studies did not report primary outcomes based on the classification. The aim of this systematic review and meta-analysis was to evaluate the primary and secondary end points and focuses on ET of SVC syndrome independently from the complexity of SVC obstruction.

In many cases of SVC syndrome, there may also be superimposed thrombosis and to address this, CDT and/or aspiration thrombectomy can be performed prior to revascularization [28]. In our meta-analysis, 11 studies reported use of CDT; however, sub-group analysis could not be performed comparing primary outcomes in SVC syndrome patients that received CDT compared to those that did not as the included studies did not report these outcomes in detail (Table 1).

The use of ET in SVC syndrome has not previously been examined in a formal meta-analysis, however, existing research demonstrating its efficacy is well-documented in a review from 2014 [28]. Since then, more studies have continued to support the use of ET as the preferred treatment option for SVC syndrome. Although the study did not meet our inclusion criteria, a recent small RCT by Takeuchi et al. examined stent placement among 32 patients with SVC or inferior vena cava occlusion. This trial demonstrated statistically significant improvements in symptom scores among the ET group as compared to control [29].

In the contemporary era, benign SVC syndrome is usually related to pacemakers and defibrillator leads and, in general, these patients have a longer life expectancy [3]. Surgical bypass was once considered the main treatment option for younger patients as it provides a durable solution. However, more recently ET has been considered first-line therapy as it does not preclude open surgical bypass in the future, and it can be combined with other treatment modalities like hybrid revascularizations. Primary patency rate for BSVC syndrome was lower compared to MSVC syndrome (75.8% vs. 86.0%), but the sample size for BSVC syndrome was much smaller (197 [9.0%] vs. 1957 [89.0%] patients). Primary patency was reported for 1301 patients for MSVC syndrome, 92 patients for BSVC syndrome, and 46 patients [2.1%] did not report etiology of SVC syndrome and not included in the analysis (Appendix 3). Nevertheless, the secondary patency rate for BSVC syndrome was acceptable (86.3%) thus supporting the paradigm that endovascular stenting can provide a durable alternative to surgical options and also does not preclude from future surgical intervention. Lower rates of primary and secondary patency in BSVC syndrome in comparison to MSVC syndrome may be related to length of follow up. Given the higher life expectancy in the BSVC group, they may have higher rates and longer durations of follow up. Studies did not confirm this finding as duration of follow up for BSVC is not separately reported*.*

Regarding the etiologies of BSVC syndrome, the most commonly reported was device-related (e.g. central venous catheters, pacemakers, defibrillator leads, hemodialysis catheters) (73.6%) followed by mediastinal fibrosis (MF) (16.2%). Other less commonly reported etiologies included post-surgical complications, radiation induced, and extrinsic compression of unknown etiology (Table 3). Even among more uncommon etiologies of SVC syndrome such as mediastinal fibrosis (MF) for which the role of ET is not well documented, evidence for its use is building. Recent studies have reported success in the use of ET to treat MF associated SVC syndrome. Although spiral vein bypass grafting has traditionally been the first-line therapy for SVC syndrome in MF, some authors suggest a multidisciplinary approach in which ET is first-line intervention and open surgical reconstruction reserved for MF associated SVC syndrome that is refractory to ET [30,31]. Sub-group analysis of the various etiologies of BSVC syndrome could not be performed in our meta-analysis as the included studies did not report the primary outcomes in this cohort.

**Table 3:** Etiology of benign SVC syndrome.

	Total [n (%)]	Rösch et al. (1992)	Kee et al. (1998)	Marcy et al. (2001)	Smayra et al. (2001)	De Gregoria Azira et al. (2003)	Mónaco et al. (2003)	Barshes et al. (2007)	Rizvi et al. (2008)	Breault et al. (2017)	Haddad et al. (2018)	Karakhanian et al. (2019)
Central venous catheter (including hemodialysis and pacemaker/defibrillator leads)	145 (73.6%)	–	12	–	9	12	2	15	19	33	33	10
Fibrous mediastinitis	32 (16.2%)	1	1	2	1	2	–	1	9	1	14	–
Post-radiotherapy	5 (2.5)	1	–	–	2	–	2	–	–	–	–	–
Post-surgical	4 (2.0)	–	1	–	2	–	–	–	–	1	–	–
Previously treated neoplasm	3 (1.5)	–	–	–	–	–	–	–	–	3	–	–
Extrinsic compression	3 (1.5)	–	–	–	–	–	–	–	–	3	–	–
Spontaneous thrombosis	1 (0.5)	–	1	–	–	–	–	–	–	–	–	–
Goiter	1 (0.5)	–	1	–	–	–	–	–	–	–	–	–
Other	3 (1.5)	–	–	–	–	–	–	–	–	3	–	–

The role of anticoagulation after revascularization of SVC has not been well studied. In the absence of notable thrombosis, the role of anticoagulation is not well established. Anticoagulation regimen for reported studies were variable and included oral anticoagulants, anti-platelet agents, or parenteral anticoagulants. Bleeding risk and outcomes based on etiology of SVC syndrome was not reported. Ten studies reported use of coumadin and two studies [32,33] reported only using Aspirin. Weighted outcomes including primary patency, secondary patency, restenosis, and recurrence were fairly similar in the two studies that used aspirin compared to other modalities. In cases of MSVC syndrome, significant thrombosis has been reported in 24% of patients and systemic anticoagulation is the standard of care, but the benefits and outcomes are not known. A large cohort study of 183 patients reported that in patients with thrombosis, anticoagulation especially at therapeutic doses is associated with higher major bleeding rates without affecting the mortality or rates of thrombosis when compared to the cohort that did not receive anticoagulation [34]. This finding therefore raises the possibility of using reduced-dose anticoagulation in patients with thrombosis. In addition, the role of CDT in relation to duration or type of anticoagulation is not known.

When SVC obstruction occurs with bilateral brachiocephalic vein involvement, relieving the obstruction in one of the occluded brachiocephalic veins is often sufficient for symptom resolution. Recanalization and stenting of one instead of both brachiocephalic veins with “kissing stents” were associated with lower rates of complications and stent thrombosis [20]. However unilateral versus bilateral stenting approach has been very operator dependent, and many operators have suggested that bilateral stenting should be considered only if the SVC diameter was >15 mm [20]. Nine studies reported use of bilateral stents but did not report outcomes compared to unilateral stent placement. Several types of stents were used in these studies and there is not enough granularity in our cumulative data to assess for differences in clinical success rates of individual stents [35,36].

Our study had several limitations. The studies which met inclusion criteria for our primary outcomes of interest mainly reported the outcomes as a cumulative finding and did not provide results in subgroups such as Stanford Doty classification, mode of anticoagulation, chronicity of presentation, and use of CDT. Given the lack of granularity and patient level data in the studies, critical subgroup analysis in these areas were not possible. Furthermore, most studies were retrospective in design with high heterogeneity for primary and secondary outcomes. To further analyze the heterogeneity, a random-effects meta-regression analysis of mean/median age and duration of follow-up did not reveal significant association with most of the end points analyzed (Appendix 4). Publication bias was noted for all outcomes in our included studies (Appendix 5). It should also be noted that over the course of this study period (1992–2020) there have been several developments in stent types and overall efficacy which is difficult to assess in this review. Moreover, there is significant variability in stent types, diameters, and lengths which can affect variables such as primary patency and restenosis. Lastly, many patients with malignant SVC syndrome have a short life expectancy post-intervention leading to decreased rates of follow-up and the ability to monitor our primary and secondary endpoints.

In summary, this meta-analysis and systematic review of 38 full-length articles demonstrate high technical success rates, low restenosis rates, and low recurrence rates following ET for SVC syndrome thus supporting the paradigm of ET as a first-line treatment of SVC syndrome for both malignant and benign etiologies. This study provides the most contemporary and cumulative evidence for the safety and efficacy of ET in the management of SVC syndrome. As the modern endovascular techniques evolve, we believe that the outcomes will continue to improve, however, there is a need for continued research on the use of ET in SVC syndrome such as larger nationwide cohort studies and RCTs.

## CRediT authorship contribution statement

**Abdul Hussain Azizi:** Formal analysis, Data curation, Writing – review & editing. **Irfan Shafi:** Formal analysis, Data curation, Writing – review & editing. **Matthew Zhao:** Formal analysis, Data curation, Writing – review & editing. **Saurav Chatterjee:** Formal analysis, Writing – review & editing. **Stephanie Clare Roth:** Formal analysis, Data curation, Writing – review & editing. **Maninder Singh:** Formal analysis, Writing – review & editing. **Vladimir Lakhter:** Formal analysis, Writing – review & editing. **Riyaz Bashir:** Formal analysis, Data curation, Writing – review & editing.

## Declaration of Competing Interest

Dr. Riyaz Bashir has an equity interest in Thrombolex Inc. All other authors have no competing interests to disclose.

## Appendix Supplementary materials

Image, application 1

Image, application 2

## References

[1] Friedman T., Quencer K.B., Kishore S.A., Winokur R.S., Madoff D.C. (2017). Malignant venous obstruction: superior vena cava syndrome and beyond. Semin Interv Radiol.

[2] Wilson L.D., Detterbeck F.C., Yahalom J. (2007). Clinical practice. Superior vena cava syndrome with malignant causes. N Engl J Med.

[3] Rice T.W., Rodriguez R.M., Light R.W. (2006). The superior vena cava syndrome: clinical characteristics and evolving etiology. Medicine.

[4] Azizi A.H., Shafi I., Shah N. (2020). Superior vena cava syndrome. JACC Cardiovasc Interv.

[5] Lanciego C., Pangua C., Chacón J.I. (2009). Endovascular stenting as the first step in the overall management of malignant superior vena cava syndrome. Am J Roentgenol.

[6] Fagedet D., Thony F., Timsit J.F. (2013). Endovascular treatment of malignant superior vena cava syndrome: results and predictive factors of clinical efficacy. Cardiovasc Intervent Radiol.

[7] de Gregorio Ariza M.A., Gamboa P., Gimeno M.J. (2003). Percutaneous treatment of superior vena cava syndrome using metallic stents. Eur Radiol.

[8] Nagata T., Makutani S., Uchida H. (2007). Follow-up results of 71 patients undergoing metallic stent placement for the treatment of a malignant obstruction of the superior vena cava. Cardiovasc Intervent Radiol.

[9] Nguyen N.P., Borok T.L., Welsh J., Vinh-Hung V. (2009). Safety and effectiveness of vascular endoprosthesis for malignant superior vena cava syndrome. Thorax.

[10] Uberoi R. (2006). Quality assurance guidelines for superior vena cava stenting in malignant disease. Cardiovasc Intervent Radiol.

[11] Barshes N.R., Annambhotla S., El Sayed H.F. (2007). Percutaneous stenting of superior vena cava syndrome: treatment outcome in patients with benign and malignant etiology. Vascular.

[12] Rizvi A.Z., Kalra M., Bjarnason H., Bower T.C., Schleck C., Gloviczki P. (2008). Benign superior vena cava syndrome: stenting is now the first line of treatment. J Vasc Surg.

[13] Hooker J.B., Hawkins B.M., Abu-Fadel M.S. (2018). Endovascular stenting in 2 patients with benign superior vena cava syndrome. Tex Heart Inst J.

[14] Kansagara D., Englander H., Salanitro A. (2011). Risk prediction models for hospital readmission: a systematic review. JAMA.

[15] Wallace B.C., Dahabreh I.J., Trikalinos T.A., Lau J., Trow P., Schmid C.H. (2012). Closing the gap between methodologists and end-users: r as a computational back-end. J Stat Softw.

[16] W. Viechtbauer Conducting meta-analyses in R with the metafor package. 2010 2010; 36(3): 48.

[17] Freeman M.F., Tukey J.W. (1950). Transformations related to the angular and the square root. Annal Math Stat.

[18] Lanciego C., Chacón J.L., Julián A. (2001). Stenting as first option for endovascular treatment of malignant superior vena cava syndrome. AJR Am J Roentgenol.

[19] Sobrinho G., Aguiar P. (2014). Stent placement for the treatment of malignant superior vena cava syndrome-a single-center series of 56 patients. Arch Bronconeumol.

[20] Dinkel H.P., Mettke B., Schmid F., Baumgartner I., Triller J., Do D.D. (2003). Endovascular treatment of malignant superior vena cava syndrome: is bilateral wallstent placement superior to unilateral placement?. J Endovasc Ther.

[21] Cho Y., Gwon D.I., Ko G.Y. (2014). Covered stent placement for the treatment of malignant superior vena cava syndrome: is unilateral covered stenting safe and effective?. Korean J Radiol.

[22] Niu S., Xu Y.S., Cheng L., Cao C. (2017). Stent insertion for malignant superior vena cava syndrome: effectiveness and long-term outcome. Radiol Med.

[23] Büstgens F.A., Loose R., Ficker J.H., Wucherer M., Uder M., Adamus R. (2017). Stent implantation for superior vena cava syndrome of malignant cause. Rofo.

[24] Kee S.T., Kinoshita L., Razavi M.K., Nyman U.R., Semba C.P., Dake M.D. (1998). Superior vena cava syndrome: treatment with catheter-directed thrombolysis and endovascular stent placement. Radiology.

[25] Smayra T., Otal P., Chabbert V. (2001). Long-term results of endovascular stent placement in the superior caval venous system. Cardiovasc Intervent Radiol.

[26] Nicholson A.A., Ettles D.F., Arnold A., Greenstone M., Dyet J.F. (1997). Treatment of malignant superior vena cava obstruction: metal stents or radiation therapy. J Vasc Interv Radiol.

[27] Cohen R., Mena D., Carbajal-Mendoza R., Matos N., Karki N. (2008). Superior vena cava syndrome: a medical emergency?. Int J Angiol.

[28] Rachapalli V., Boucher L.M. (2014). Superior vena cava syndrome: role of the interventionalist. Can Assoc Radiol J.

[29] Takeuchi Y., Arai Y., Sone M. (2019). Evaluation of stent placement for vena cava syndrome: phase II trial and phase III randomized controlled trial. Support Care Cancer.

[30] Sfyroeras G.S., Antonopoulos C.N., Mantas G. (2017). A review of open and endovascular treatment of superior vena cava syndrome of benign aetiology. Eur J Vasc Endovasc Surg.

[31] Deshwal H., Ghosh S., Magruder K., Bartholomew J.R., Montgomery J., Mehta A.C. (2020). A review of endovascular stenting for superior vena cava syndrome in fibrosing mediastinitis. Vasc Med.

[32] Marcy P.Y., Magne N., Bentolila F., Drouillard J., Bruneton J.N., Descamps B. (2001). Superior vena cava obstruction: is stenting necessary?. Support Care Cancer.

[33] Andersen P.E., Duvnjak S. (2014). Palliative treatment of superior vena cava syndrome with nitinol stents. Int J Angiol.

[34] Ratzon R., Tamir S., Friehmann T. (2019). Thrombosis, anticoagulation and outcomes in malignant superior vena cava syndrome. J Thromb Thrombolysis.

[35] Nguyen N.P., Borok T.L., Welsh J., Vinh-Hung V. (2009). Safety and effectiveness of vascular endoprosthesis for malignant superior vena cava syndrome. Thorax.

[36] Gwon D.I., Ko G.Y., Kim J.H., Shin J.H., Yoon H.K., Sung K.B. (2013). Malignant superior vena cava syndrome: a comparative cohort study of treatment with covered stents versus uncovered stents. Radiology.

[37] Rösch J., Uchida B.T., Hall L.D. (1992). Gianturco-Rösch expandable *Z*-stents in the treatment of superior vena cava syndrome. Cardiovasc Interv Radiol.

[38] Oudkerk M., Kuijpers T.J., Schmitz P.I., Loosveld O., de Wit R. (1996). Self-expanding metal stents for palliative treatment of superior vena caval syndrome. Cardiovasc Interv Radiol.

[39] Tanigawa N., Sawada S., Mishima K. (1998). Clinical outcome of stenting in superior vena cava syndrome associated with malignant tumors. comparison with conventional treatment. Acta Radiol.

[40] Thony F., Moro D., Witmeyer P. (1999). Endovascular treatment of superior vena cava obstruction in patients with malignancies. Eur Radiol.

[41] Miller J.H., McBride K., Little F., Price A. (2000). Malignant superior vena cava obstruction: stent placement via the subclavian route. Cardiovasc Interv Radiol.

[42] Courtheoux P., Alkofer B., Al Refaï M., Gervais R., Le Rochais J.P., Icard P. (2003). Stent placement in superior vena cava syndrome. Ann Thorac Surg.

[43] García Mónaco R., Bertoni H., Pallota G. (2003). Use of self-expanding vascular endoprostheses in superior vena cava syndrome. Eur J Cardiothorac Surg.

[44] Urruticoechea A., Mesia R., Dominguez J. (2004). Treatment of malignant superior vena cava syndrome by endovascular stent insertion. experience on 52 patients with lung cancer. Lung Cancer.

[45] Da Ines D., Chabrot P., Cassagnes L. (2008). Endovascular treatment of SVC syndrome from neoplastic origin: a review of 34 cases. J Radiol.

[46] Berna P., Bagan P., Renard C., Auquier M., Remond A., Riquet M. (2008). Pulmonary malignant superior vena cava obstruction: endovascular stent therapy. Rev Pneumol Clin.

[47] Lanciego C., Pangua C., Chacon J.I. (2009). Endovascular stenting as the first step in the overall management of malignant superior vena cava syndrome. AJR Am J Roentgenol.

[48] Maleux G., Marchal P., Palmers M. (2010). Catheter-directed thrombolytic therapy for thoracic deep vein thrombosis is safe and effective in selected patients with and without cancer. Eur Radiol.

[49] Leung S.T., Sung T.H., Wan A.Y., Leung K.W., Kan W.K. (2015). Endovascular stenting in the management of malignant superior vena cava obstruction: comparing safety, effectiveness, and outcomes between primary stenting and salvage stenting. Hong Kong Med J.

[50] Mokry T., Bellemann N., Sommer C.M. (2015). Retrospective study in 23 patients of the self-expanding sinus-XL stent for treatment of malignant superior vena cava obstruction caused by non-small cell lung cancer. J Vasc Interv Radiol.

[51] Calsina Juscafresa L., Gil Bazo I., Grochowicz L. (2017). Endovascular treatment of malignant superior vena cava syndrome secondary to lung cancer. Hosp Pract.

[52] Breault S., Doenz F., Jouannic A.-.M., Qanadli S.D. (2017). Percutaneous endovascular management of chronic superior vena cava syndrome of benign causes: long-term follow-up. Eur Radiol.

[53] Haddad M.M., Simmons B., McPhail I.R. (2018). Comparison of covered versus uncovered stents for benign superior vena cava (SVC) obstruction. Cardiovasc Interv Radiol.

[54] Anton S., Oechtering T., Stahlberg E. (2018). Endovascular stent-based revascularization of malignant superior vena cava syndrome with concomitant implantation of a port device using a dual venous approach. Support Care Cancer.

[55] Wang Z.S., Li C.W., Li J.X., Wu W.J., Li Y., Shi J.G. (2019). Covered versus uncovered stent insertion for malignant superior vena cava obstruction. Minim Invasive Ther Allied Technol.

[56] Karakhanian W.K., Karakhanian W.Z., Belczak S.Q. (2019). Superior vena cava syndrome: endovascular management. J Vasc Bras.

[57] McDevitt J.L., Goldman D.T., Bundy J.J. (2020). Gianturco *Z*-stent placement for the treatment of chronic central venous occlusive disease: implantation of 208 stents in 137 symptomatic patients. Diagn Interv Radiol.

[58] Irace L., Martinelli O., Gattuso R. (2021). The role of self-expanding vascular stent in superior vena cava syndrome for advanced tumours. Ann R Coll Surg Engl.

