# Supplementary material for: Endovascular therapy for superior vena cava syndrome: A systematic review and meta-analysis
Source: eClinicalMedicine. 2021 Jun 28;37:100970. doi: 10.1016/j.eclinm.2021.100970 (PMC8343254; doi:10.1016/j.eclinm.2021.100970)
Supplement: Supplementary file 1 [file mmc1.docx]

Appendix 1: PICOTS Elements

Appendix 2: Search Strategy Characteristics

Appendix 3: Sensitivity Analysis of Malignant SVC Syndrome and Benign SVC Syndrome

- Figure 1A: Primary Patency of Malignant SVC (MSVC) Syndrome
- Figure 1B: Primary Patency of Benign SVC (BSVC) Syndrome
- Figure 1C: Summary Table of Primary Patency for MSVC and BSVC Syndrome
- Figure 2A: Secondary Patency of Malignant SVC (MSVC) Syndrome
- Figure 2B: Secondary Patency of Benign SVC (BSVC) Syndrome
- Figure 2C: Summary Table of Secondary Patency for MSVC and BSVC Syndrome

Appendix 4: Meta-regression of High Heterogeneity

Appendix 5: Funnel Plots

- Figure 1: Technical Success Funnel Plot for Publication Bias
- Figure 2: Restenosis Funnel Plot for Publication Bias
- Figure 3: Recurrence Funnel Plot for Publication Bias
- Figure 4: Primary Patency Funnel Plot for Publication Bias
- Figure 5: Secondary Patency Funnel Plot for Publication Bias
