# Supplementary material for: Endovascular therapy for superior vena cava syndrome: A systematic review and meta-analysis
Source: eClinicalMedicine. 2021 Jun 28;37:100970. doi: 10.1016/j.eclinm.2021.100970 (PMC8343254; doi:10.1016/j.eclinm.2021.100970)

Appendix 1: PICOTS Elements

| **PICOTS Element** | **Inclusion Criteria** | **Exclusion Criteria** |
| --- | --- | --- |
| Patient Population | • Adults over the age of 18 with SVC syndrome, including benign and malignant SVC syndrome | • Patients younger than 18 years of age |
| Intervention/  Comparator | • Endovascular stenting for the treatment of SVC syndrome |  |
| Outcomes | • Technical success rate  • Restenosis rate  • SVC recurrence rate  • Stent Patency   - Primary Patency - Secondary |  |
| Timing | • Any duration of follow-up to be included |  |
| Setting | • All clinical settings, including inpatient and outpatient |  |
| Study Design | • Original data  • Study size n=20 or greater  • Randomized controlled trials, prospective, and retrospective observational studies | • Case reports  • Case series including less than 20 patients  • Study did not include any outcomes of interest |
| Publications | • Published from inception of databases to September 25, 2020 | • Study patients already examined in previously included study |

Appendix 2: Search Strategy Characteristics

PubMed (NLM)

| (“vena cava*”[tiab] OR "Venae Cavae"[Mesh] OR "Vena Cava, Superior"[Mesh] OR "Superior Vena Cava Syndrome"[Mesh] OR SVC[tiab] OR SVCS[tiab] OR “venae cava*”[tiab] OR “superior vena cava*”[tiab] OR “superior venae cava*”[tiab] OR “SVC syndrom*”[tiab] OR “malignant superior vena cava*”[tiab] OR “superior vena cava syndrom*”[tiab] OR “SVC obstruct*”[tiab]) AND ("Stenosis, Pulmonary Vein"[Mesh] OR "Constriction, Pathologic"[Mesh] OR stenosis*[tiab] OR “pulmonary vein stenosis*”[tiab] OR “pulmonary venous stenosis*”[tiab] OR “pathologic constrict*”[tiab] OR abnormal*[tiab] OR narrow*[tiab] OR occlusion*[tiab] OR strictur*[tiab] OR constrict*[tiab] OR “vascular patenc*”[tiab] OR patenc*[tiab] OR thrombos*[tiab] OR obstruct*[tiab] OR compress*[tiab] OR interrupt*[tiab] OR restenosis*[tiab]) AND ((stent*[tiab] OR "Stents"[Mesh] OR angioplast*[tiab] OR endovascul*[tiab] OR "Endovascular Procedures"[Mesh] OR “endovascular therap*”[tiab] OR “Endovascular treat*”[tiab] OR "Mediastinal Fibrosis"[Supplementary Concept] OR "Multifocal fibrosclerosis"[Supplementary Concept] OR “fibrosis mediastinal*”[tiab] OR "Mediastinitis"[Mesh] OR "Mediastinal Diseases"[Mesh] OR "Blood Vessel Prosthesis Implantation"[Mesh] OR "Blood Vessel Prosthesis"[Mesh] OR “blood vessel prosthesis*”[tiab] OR mediastinit*[tiab] OR “endovascular stent*”[tiab] OR “bilateral placement*”[tiab] OR “unilateral placement*”[tiab] OR “stent placement*”[tiab] OR “covered stent*”[tiab] OR “catheter directed thrombolysis*”[tiab] OR “uncovered stent*”[tiab] OR wallstent*[tiab] OR bcv[tiab] OR svc[tiab] OR ("Stents"[Mesh] AND endovascul*[tiab]) OR (stent*[tiab] AND endovascul*[tiab])) NOT ((animals[MeSH Terms]) NOT ((animals[MeSH Terms]) AND (humans[MeSH Terms]))) AND 1988/01/01:2030/01/01[dp] |
| --- |

Embase (Elsevier)

| ('vena* cava*':ti,ab OR svc:ti,ab OR svcs:ti,ab OR 'superior vena* cava*':ti,ab OR 'svc syndrom*':ti,ab OR 'malignant superior vena* cava*':ti,ab OR ((malignant* NEAR/3 'superior vena* cava*'):ti,ab) OR 'superior vena* cava syndrom*':ti,ab OR 'svc obstruct*':ti,ab) AND (stenosis*:ti,ab OR 'stenosis'/exp OR 'stenosis, occlusion and obstruction'/exp OR 'pulmonar* vein* stenosis*':ti,ab OR 'pulmonar* venous* stenosis*':ti,ab OR 'pathologic* constrict*':ti,ab OR abnormal*:ti,ab OR narrow*:ti,ab OR occlusion*:ti,ab OR strictur*:ti,ab OR constrict*:ti,ab OR 'vascular* patenc*':ti,ab OR patenc*:ti,ab OR thrombos*:ti,ab OR obstruct*:ti,ab OR compress*:ti,ab OR interrupt*:ti,ab OR restenosis*:ti,ab) AND ((stent*:ti,ab OR 'stent'/exp OR angioplast*:ti,ab OR endovascul*:ti,ab) AND ((endovascular* NEAR/3 stent*):ti,ab) OR 'endovascular therap*':ti,ab OR 'endovascul* treat*':ti,ab OR 'fibros* mediastinal*':ti,ab OR 'blood* vessel* prosthesis*':ti,ab OR mediastinit*:ti,ab OR 'mediastinal fibrosis'/exp OR 'mediastinitis'/exp OR 'endovascul* stent*':ti,ab OR ((placement* NEAR/3 stent*):ti,ab) OR 'bilateral placement*':ti,ab OR 'unilateral placement*':ti,ab OR 'stent* placement*':ti,ab OR 'cover* stent*':ti,ab OR 'catheter* direct* thrombolysis*':ti,ab OR 'uncover* stent*':ti,ab OR wall?stent*:ti,ab OR bcv:ti,ab OR svc:ti,ab) AND [1988-2021]/py |
| --- |

Cochrane Central (Wiley)

| (“vena cava*”:ti,ab,kw OR SVC:ti,ab,kw OR SVCS:ti,ab,kw OR “venae cava*”:ti,ab,kw OR “superior vena cava*”:ti,ab,kw OR “superior venae cava*”:ti,ab,kw OR “SVC syndrom*”:ti,ab,kw OR “malignant superior vena cava*”:ti,ab,kw OR “superior vena cava syndrom*”:ti,ab,kw OR “SVC obstruct*”:ti,ab,kw) AND (stenosis*:ti,ab,kw OR “pulmonary vein stenosis*”:ti,ab,kw OR “pulmonary venous stenosis*”:ti,ab,kw OR “pathologic constrict*”:ti,ab,kw OR abnormal*:ti,ab,kw OR narrow*:ti,ab,kw OR occlusion*:ti,ab,kw OR strictur*:ti,ab,kw OR constrict*:ti,ab,kw OR “vascular patenc*”:ti,ab,kw OR patenc*:ti,ab,kw OR thrombos*:ti,ab,kw OR obstruct*:ti,ab,kw OR compress*:ti,ab,kw OR interrupt*:ti,ab,kw OR restenosis*:ti,ab,kw) AND (stent*:ti,ab,kw OR angioplast*:ti,ab,kw OR endovascul*:ti,ab,kw OR “endovascular therap*”:ti,ab,kw OR “Endovascular treat*”:ti,ab,kw OR “fibrosis mediastinal*”:ti,ab,kw OR “blood vessel prosthesis*”:ti,ab,kw OR mediastinit*:ti,ab,kw OR “endovascular stent*”:ti,ab,kw OR “bilateral placement*”:ti,ab,kw OR “unilateral placement*”:ti,ab,kw OR “stent placement*”:ti,ab,kw OR “covered stent*”:ti,ab,kw OR “catheter directed thrombolysis*”:ti,ab,kw OR “uncovered stent*”:ti,ab,kw OR wallstent*:ti,ab,kw OR bcv:ti,ab,kw OR svc:ti,ab,kw) with Publication Year from 1988 to 2021, in Trials |
| --- |

Clinicaltrials.gov

| Vena Cava AND stent \| Completed Studies |
| --- |

TRIP Database

| (title:vena* cava* OR "venous cava*")(title:stent* OR "endovascular stent*") |
| --- |

Appendix 3: Sensitivity Analysis

Figure 1A: Primary Patency of Malignant SVC (MSVC) Syndrome


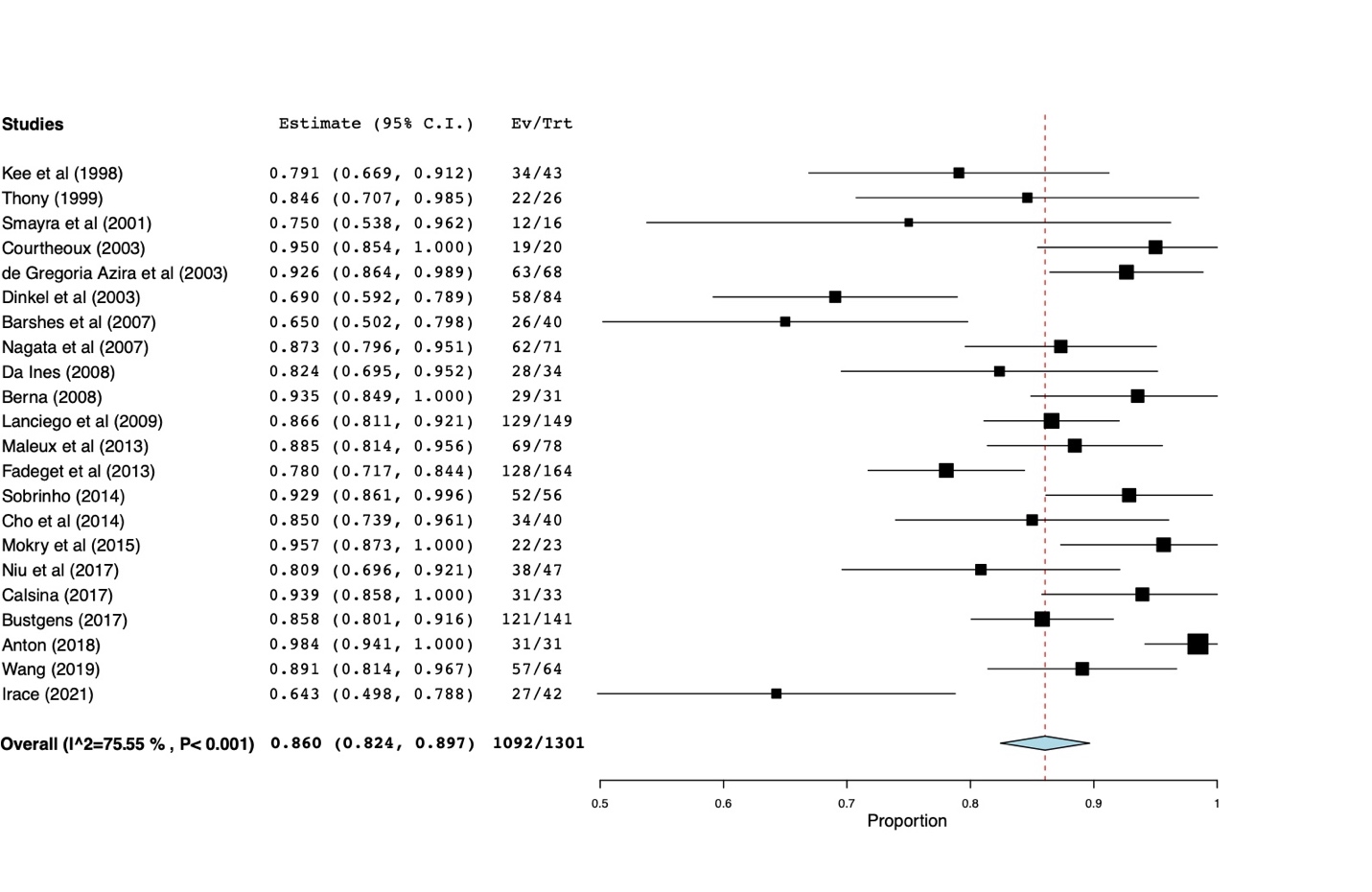


Figure 1B: Primary Patency of Benign SVC (BSVC) Syndrome


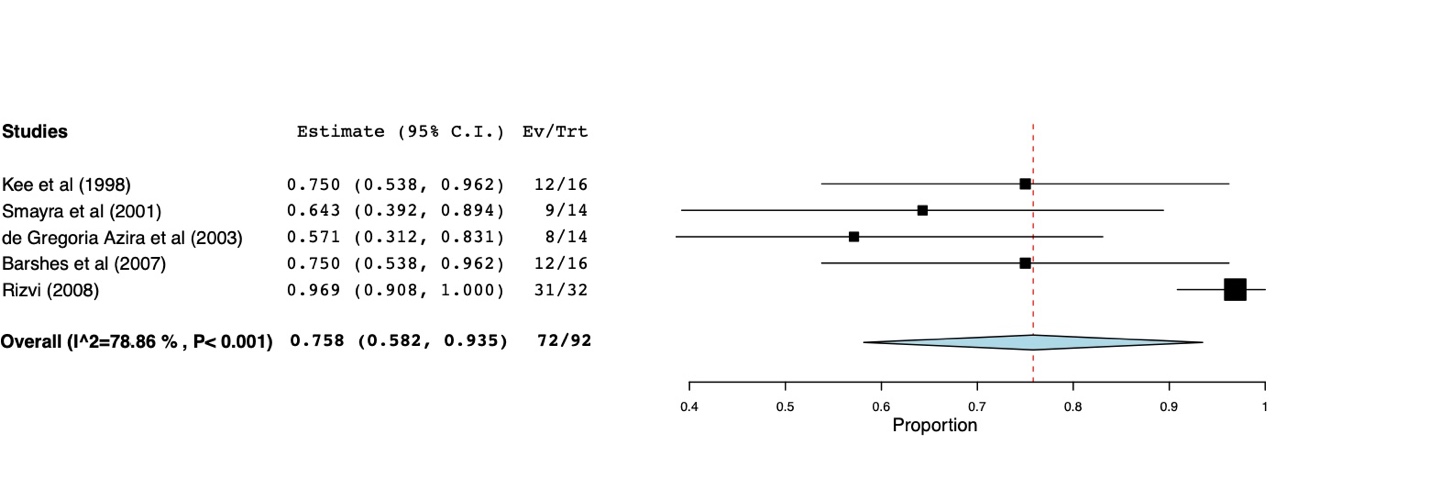


Figure 1C: Summary Table of Primary Patency for MSVC and BSVC Syndrome

|  | Overall | Lower limit | Upper limit | P value |
| --- | --- | --- | --- | --- |
| Primary Patency MSVC | 0.860 | 0.824 | 0.897 | <0.001 |
|  | Q(df=21) = 86 | I^2 = 75.6 |  |  |
| Primary Patency BSVC | 0.758 | 0.582 | 0.935 | <0.001 |
|  | Q(df=4) = 19 | I^2 = 78.9 |  |  |

Figure 2A: Secondary Patency of Malignant SVC (MSVC) Syndrome


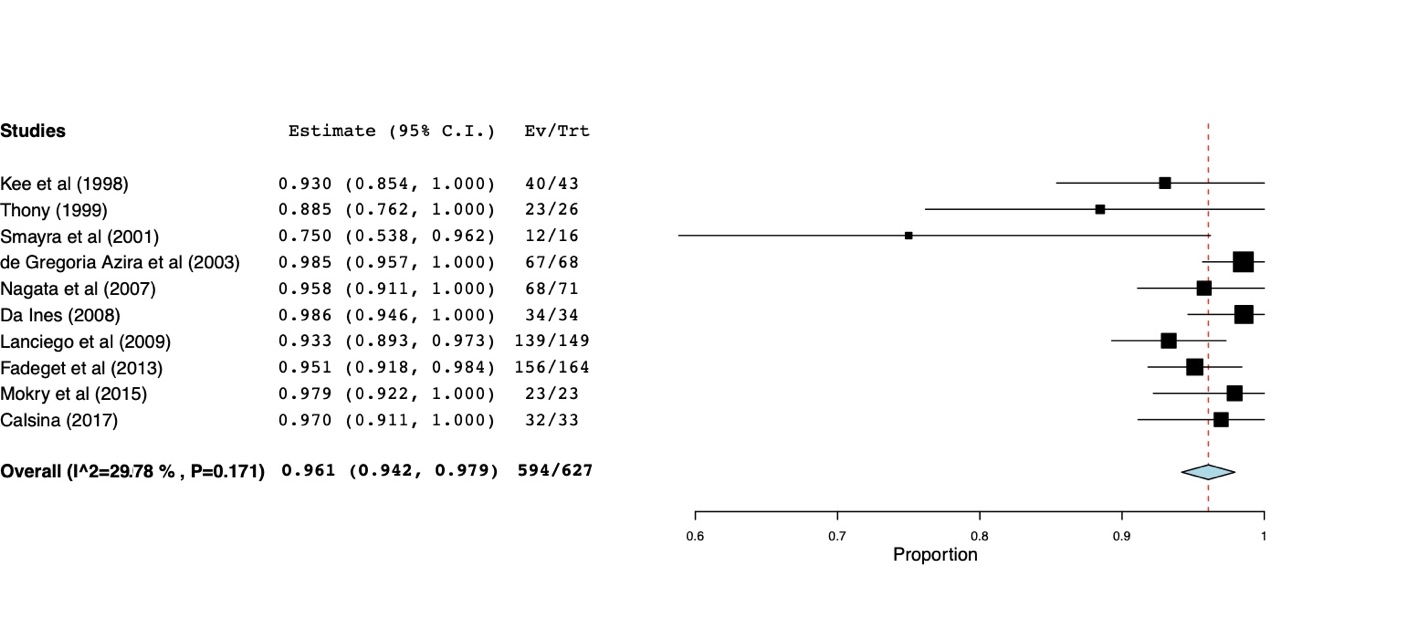


Figure 2B: Secondary Patency of Benign SVC (BSVC) Syndrome


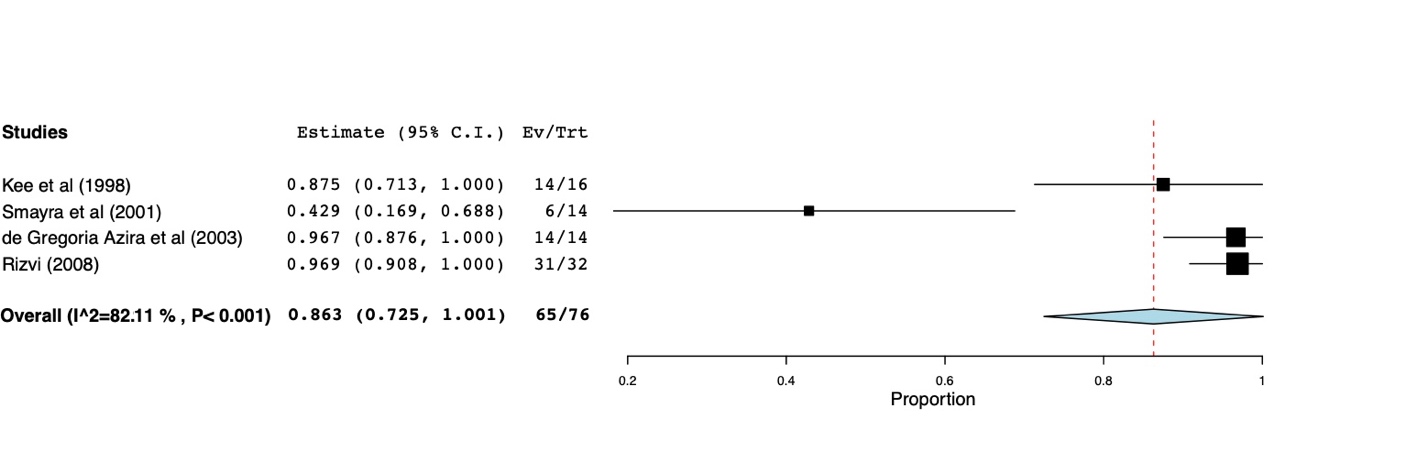


Figure 2C: Summary Table of Secondary Patency for MSVC and BSVC Syndrome

|  | Overall | Lower limit | Upper limit | P value |
| --- | --- | --- | --- | --- |
| Secondary Patency MSVC | 0.961 | 0.942 | 0.979 | <0.001 |
|  | Q(df=9) = 13 | I^2 = 29.8 |  |  |
| Secondary Patency BSVC | 0.863 | 0.725 | 1.001 | <0.001 |
|  | Q(df=3) = 17 | I^2 = 82.1 |  |  |

**Appendix 4:** Meta-regression of High Heterogeneity

**Appendix 4**- Several outcomes (restenosis, recurrence, primary patency, and secondary patency) was associated with a high heterogeneity. To further assess the high heterogeneity, mean/median age of the participants in each included study was used to perform a random-effects meta-regression analysis and it did not reveal any significant association with the individual end-points to explain the high heterogeneity. Similarly, duration of follow-up was evaluated in a random-effects model for meta-regression analysis which revealed significant association with the heterogeneity with the restenosis outcome, but not with the other end-points evaluated.

**Appendix 5: Funnel Plots for Publication Bias**

**Figure 1: Technical Success Funnel Plot for Publication Bias**

Fixed effects (inverse variance)

Pooled proportion = 0.983417 (95% CI = 0.977075 to 0.988749) Non-combinability of studies

Cochran Q = 91.721191 (df = 34) P < 0.0001

Moment-based estimate of between studies variance = 0.03269 I_2_ (inconsistency) = 62.9% (95% CI = 43.9% to 73.5%)

Random effects (DerSimonian-Laird)

Pooled proportion = 0.985699 (95% CI = 0.974991 to 0.993463) Bias indicators

Begg-Mazumdar: Kendall's -0.743197 P < 0.0001

Egger: bias = -0.802828 (95% CI = -1.501114 to -0.104541) P = 0.0255

Harbord: bias = 3.839081 (92.5% CI = 0.694812 to 6.98335) P = 0.0316


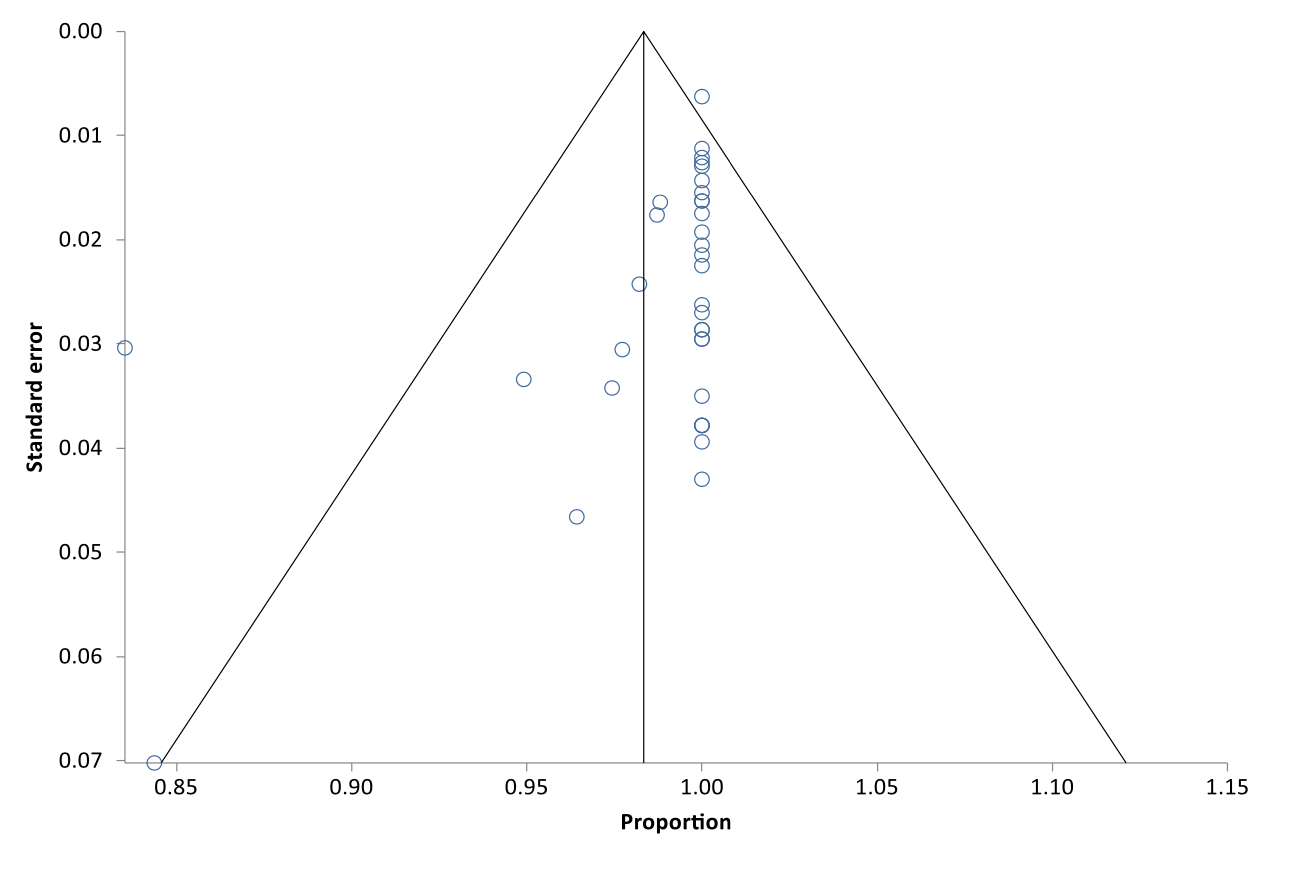


**Figure 2: Restenosis Funnel Plot for Publication Bias**

Fixed effects (inverse variance)

Pooled proportion = 0.122926 (95% CI = 0.107859 to 0.138833) Non-combinability of studies=

Cochran Q = 56.903676 (df = 30) P = 0.0021

Moment-based estimate of between studies variance = 0.016296 I_2_ (inconsistency) = 47.3% (95% CI = 12.1% to 64.6%)

Random effects (DerSimonian-Laird)

Pooled proportion = 0.121261 (95% CI = 0.100142 to 0.1441) Bias indicators

Begg-Mazumdar: Kendall's 0.268817 P = 0.0343

Egger: bias = 1.765873 (95% CI = 0.216445 to 3.315301) P = 0.0269

Harbord: bias = -0.827894 (92.5% CI = -2.790786 to 1.134998) P = 0.4423


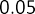

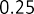

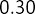

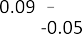

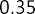

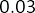

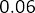

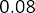


**Figure 3: Recurrence Funnel Plot for Publication Bias**

Fixed effects (inverse variance)

Pooled proportion = 0.124665 (95% CI = 0.109244 to 0.140952)

Non-combinability of studies

Cochran Q = 94.319605 (df = 30) P < 0.0001

Moment-based estimate of between studies variance = 0.040399 I_2_ (inconsistency) = 68.2% (95% CI = 51.7% to 77.2%)

Random effects (DerSimonian-Laird)

Pooled proportion = 0.118707 (95% CI = 0.091654 to 0.148734) Bias indicators

Begg-Mazumdar: Kendall's 0.310345 P = 0.015

Egger: bias = 2.655319 (95% CI = 1.261385 to 4.049254) P = 0.0005

Harbord: bias = -1.495439 (92.5% CI = -3.67818 to 0.687302) P = 0.2158


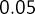

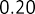

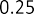

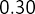

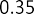

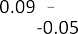

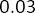

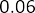

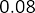


**Figure 4: Primary Patency Funnel Plot for Publication Bias**

Fixed effects (inverse variance)

Pooled proportion = 0.811089 (95% CI = 0.78995 to 0.831356) Non-combinability of studies

Cochran Q = 135.125792 (df = 21) P < 0.0001

Moment-based estimate of between studies variance = 0.088914 I_2_ (inconsistency) = 84.5% (95% CI = 77.7% to 88.4%)

Random effects (DerSimonian-Laird)

Pooled proportion = 0.815841 (95% CI = 0.75924 to 0.866381) Bias indicators

Begg-Mazumdar: Kendall's -0.367965 P = 0.014

Egger: bias = -4.31769 (95% CI = -7.386321 to -1.249059) P = 0.0082

Harbord: bias = 0.016962 (92.5% CI = -3.70886 to 3.742783) P = 0.9933


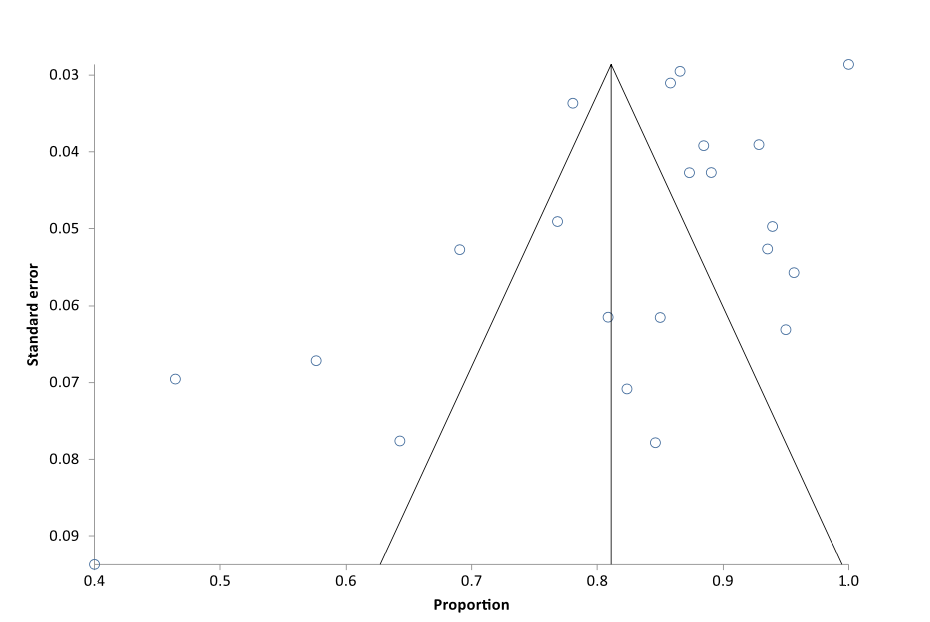


**Figure 5: Secondary Patency Funnel Plot for Publication Bias**

Fixed effects (inverse variance)

Pooled proportion = 0.901204 (95% CI = 0.877592 to 0.922536) Non-combinability of studies

Cochran Q = 87.654502 (df = 9) P < 0.0001

Moment-based estimate of between studies variance = 0.137175 I_2_ (inconsistency) = 89.7% (95% CI = 83.6% to 92.9%)

Random effects (DerSimonian-Laird)

Pooled proportion = 0.887345 (95% CI = 0.798243 to 0.952915) Bias indicators

Begg-Mazumdar: Kendall's -0.511111 P = 0.0286

Egger: bias = -4.228888 (95% CI = -7.871596 to -0.58618) P = 0.0281

Harbord: bias = -3.329924 (92.5% CI = -11.013908 to 4.35406) P = 0.4012


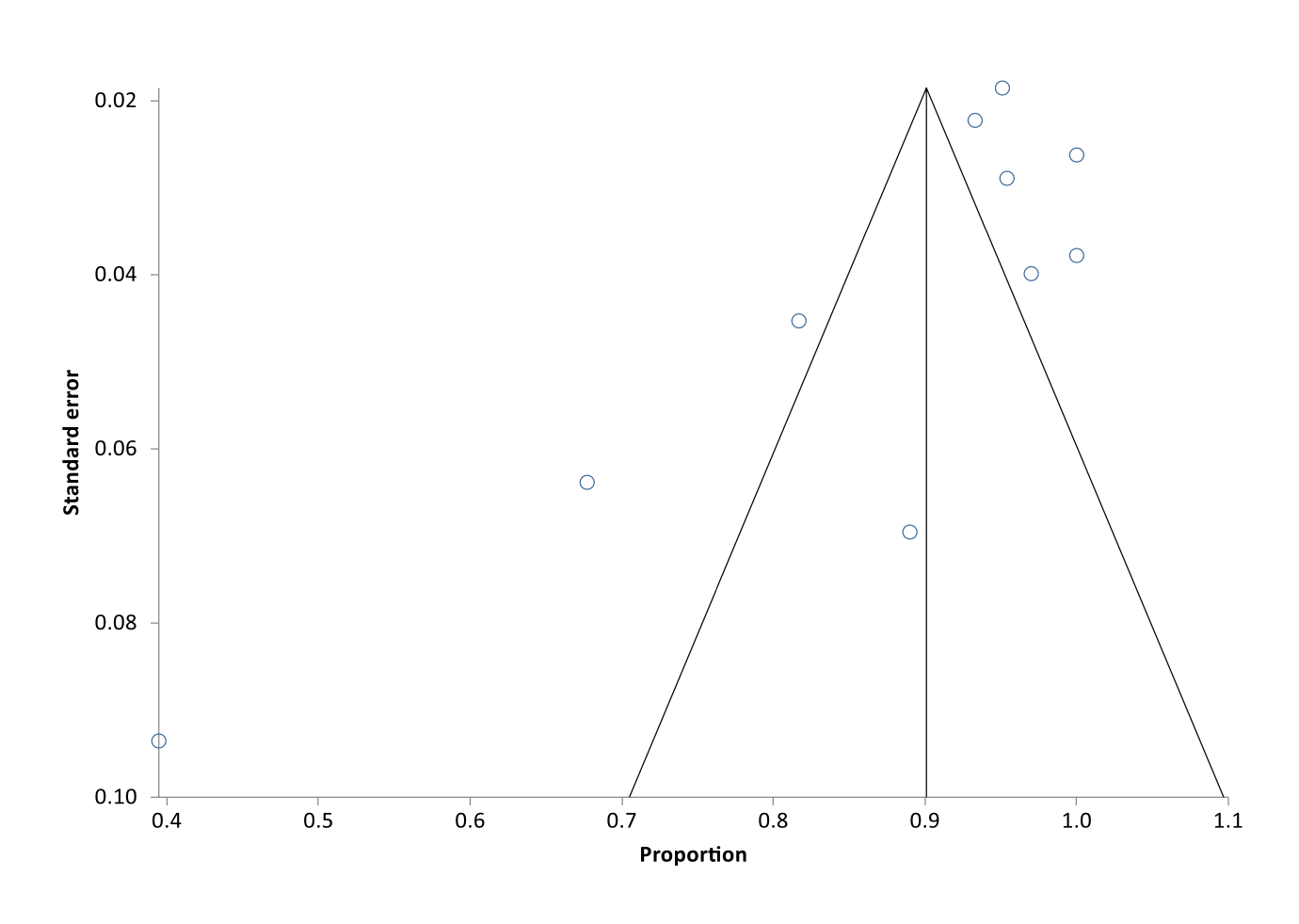

Supplement: Supplementary file 2 [file mmc2.docx]
